# Overlap and Differences of Autism and ADHD: Digital Phenotyping of Movement and Communication During Development

**DOI:** 10.1101/2025.10.20.682864

**Published:** 2025-10-20

**Authors:** Aimar Silvan, Adriana Di Martino, Michael P. Milham, Lucas C. Parra, Jens Madsen

**Affiliations:** 1 Department of Biomedical Engineering, City College of New York, New York, NY, 10031, USA; 2 Child Mind Institute, New York, NY, 10022, USA

## Abstract

Attention-Deficit/Hyperactivity Disorder (ADHD) and Autism Spectrum Disorder (ASD) often co-occur, which complicates diagnosis across development. Here, we test whether automatic analysis of naturalistic video can disentangle shared from distinct behavioral signatures of the two disorders. We analyzed videos of 2,341 youths (ages 5–22) from a community sample while they described an emotive short film. Multivariate models revealed that language deficits previously attributed to ADHD were largely explained by age. Difficulties in understanding and recalling a social narrative, sometimes attributed to ADHD, were uniquely predicted by ASD. Increased motor activity was a specific marker of the hyperactive-impulsive domain of ADHD. Conversely, ASD showed structurally intact language but significant impairments in narrative ability and perspective-taking, coupled with a unique vocal profile of higher pitch, intensity, and altered voice quality. These findings suggest that despite substantial comorbidity, ADHD and ASD exhibit separable behavioral profiles that can be measured objectively at scale.

## Introduction

Attention-Deficit/Hyperactivity Disorder (ADHD) and Autism Spectrum Disorder (ASD) affect a significant portion of youth and often create long-term challenges in education, social relationships, and overall quality of life^1–4^. Diagnostic criteria define ADHD as persistent, significantly impairing inattention and/or hyperactivity across multiple settings, while ASD is characterized by impairments in social reciprocity skills accompanied by restricted, repetitive behaviors and interests^5^. However, this distinction is often blurred by substantial clinical overlap. The disorders frequently co-occur, and individuals with either condition exhibit heterogeneity in their symptoms^6–12^, a complexity echoed by shared genetic risk factors^13–16^ and converging atypicalities in large-scale brain networks^17–22^.

Current diagnostic assessments for ASD and ADHD predominantly rely on clinical observation and informant reports, methods that are inherently subjective and resource-intensive but also struggle to reliably differentiate the two disorders or account for their comorbidity^23–25^. This reliance on manual, expert-driven analysis has traditionally limited research to smaller, often narrowly defined samples that do not capture the vast heterogeneity inherent in ADHD and ASD in more natural settings. This highlights the need for objective, quantifiable, and scalable markers derived from naturalistic behavior. Such markers may more clearly reveal the complex interplay of symptoms in ASD and ADHD as they manifest in real-world interactions.

Effective communication depends on both structural and pragmatic language skills. Structural language encompasses fundamental aspects of language production, such as vocabulary and syntax, and pragmatic language involves using these skills appropriately in social contexts to convey meaning, maintain topic coherence, and respond relevantly^26^. While deficits in social communication are a defining characteristic of ASD, they are not considered a core diagnostic feature of ADHD^5^. Despite this, studies using informant-based assessments suggest a significant degree of overlap, with both groups demonstrating difficulties in both pragmatic and structural language^27–29^.

Another aspect that is central to successful communication in a social setting is perspective-taking, often described as Theory of Mind (ToM), i.e., the ability to infer the mental and emotional states of others^30^, which are also essential for discourse and narrative comprehension and construction^31^. Difficulties in perspective-taking have long been associated with ASD^30^, but the investigation of such abilities has progressed independently for ADHD and ASD. Natural language processing (NLP) as an objective tool has identified distinct linguistic patterns in smaller-scale ASD studies, such as repetitive speech^31^, and atypical narratives^31,32^, while separate studies have linked ADHD to narrative disorganization^33^. To date, studies using automated methods have typically used small sample sizes with fewer than fifty participants^34^. This limits the power to identify meaningful and replicable linguistic and socio-cognitive profiles that scale to larger cohorts in naturalistic settings. Additionally, conducting analyses of youth with ASD or ADHD separately has prevented the delineation of shared versus distinct domains. This study addresses these gaps by exploring both language (structural and pragmatic) and higher-order social-cognitive (memory, narrative comprehension and construction, perspective-taking) abilities to develop a detailed model of impairment in both ADHD and ASD.

Aside from difficulties in social communication, atypical prosody is a commonly reported clinical feature of ASD^32^. While early research using subjective ratings struggled to conceptually define the clinically observed “unusual” prosody in ASD^33^, recent systematic reviews consistently identify higher mean pitch and greater pitch variability as features of ASD^34^. However, for other acoustic features like vocal intensity, speech rate, and voice quality, findings have remained inconsistent, likely due to the small sample sizes, diversity of methods, and clinical heterogeneity across studies^34,35^. Importantly, the potential influence of comorbid ADHD, which may also be associated with altered speech prosody^36,37^, remains poorly understood. Although recent automated methods have shown promise for ASD severity identification or ASD diagnosis based on vocal patterns^38–40^, their application to small, constrained samples leaves a gap in our ability to isolate a true, disorder-specific vocal signature for either condition.

Motor behavior offers another important domain for differentiating these disorders. Research in ASD has characterized a wide range of atypical motor behaviors^41,42^. In contrast, objective motor analysis in ADHD has remained comparatively sparse, focusing primarily on global activity levels via actigraphy^43^. Additionally, to date, no study has used the same objective movement measures to directly compare ADHD and ASD while simultaneously modeling their high rate of comorbidity.

Further complicating matters, language, speech, and motor coordination develop rapidly with age, and symptom presentations can shift^44^. For instance, hyperactivity in ADHD often decreases while inattention persists into adolescence^45^. Similarly, the co-occurrence of ASD and ADHD symptoms emerges in middle childhood and appears to peak during adolescence, a crucial and understudied period of social and cognitive change^44^. Without rigorously modeling the effect of age, along with other key sources of variability in ASD and ADHD, such as different prevalence in boys and girls^46,47^, or wide ranges of cognitive abilities as measured by IQ^48,49^, most behavioral markers may simply reflect demographic and cognitive differences or typical developmental trajectories rather than underlying psychopathology.

We address these gaps by leveraging artificial intelligence (AI) to extract objective markers of language, speech, and movement from audio and video recordings of conversations from the Healthy Brain Network cohort (HBN)^50^. This large community sample includes youths with a variety of mental health and neurodevelopmental conditions, including ADHD and ASD, along with neurotypical controls. This large-scale approach links computationally-derived behaviors to gold-standard, consensus-based clinical diagnoses, their differential presentation, and co-occurrence, overcoming the limitations of smaller studies that rely on subjective instruments. We test two questions central to the field: (1) Do ASD and ADHD show distinct or overlapping behavioral signatures? and (2) How are these signatures shaped by development, sex, and IQ? This work represents the first large-scale validation of digital phenotyping to deconstruct the behavioral heterogeneity of these two overlapping neurodevelopmental disorders.

## Results

### A large-scale developmental cohort for digital phenotyping

**Figure 1.**
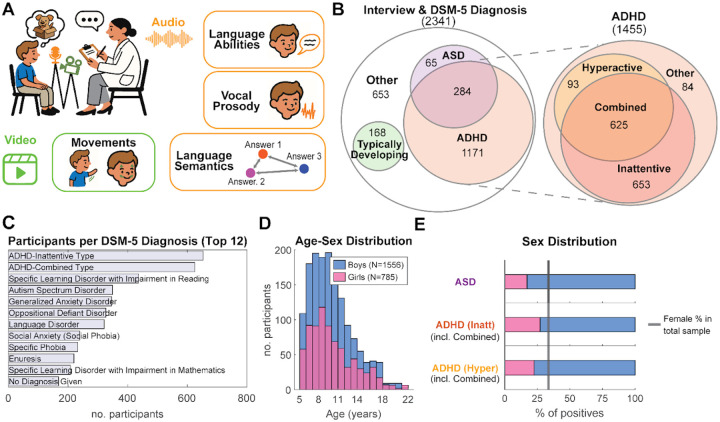
Overview of the study cohort. **A)** Using AI for objective digital phenotyping. Video recordings were processed to quantify body and facial movements, while audio was transcribed and analyzed to extract metrics of language structure, semantic content, and vocal prosody. **B)** Venn diagram showing the distribution and overlap of the primary diagnostic groups. **C)** The 12 most common clinician-assigned DSM-5 diagnoses in the sample. **D)** Age and sex distribution of the full cohort (N=2,341). **E)** Sex distribution within the children with ASD and/or ADHD diagnostic, ADHD-Combined are here considered positive in both hyperactive and inattentive diagnostic status. The black line indicates chance occurrence based on the sex ratio in the cohort.

To measure naturalistic behaviors, 2,341 participants (aged 5–22) of the HBN cohort were video-recorded during a semi-structured interview after watching “The Present”, an emotive animated short film. During the interview participants are asked identical questions (Extended Data Table S1 & Extended Data Fig. S1) and the adult interviewer follows up with encouraging prompts when necessary to elicit responses. This conversation serves as a social-emotional probe, eliciting responses across broad domains, including narrative production and emotional expression, while also probing social-cognitive functions relevant to ASD, such as narrative comprehension and perspective-taking (Theory of Mind) (see Extended Data Table S2 for an overview of related measures). We used state-of-the-art computational tools to derive objective, quantitative metrics from the video-recorded interviews, covering expressive language, vocal prosody, as well as facial and body movements (Fig. 1A).

The participant cohort was diagnostically rich and clinically complex (Fig. 1B, C & Table 1). It included 1,455 youths with clinician-confirmed ADHD DSM-5 diagnoses. Among these, 653 had an inattentive presentation, 93 a hyperactive-impulsive presentation, and 625 met criteria for a combined presentation. A subset (N = 84) did not meet full diagnostic criteria (categorized in the DSM-5 as ADHD other/unspecified) and were excluded from analysis. The sample also included 349 participants with mild to moderate ASD (see Extended Data Fig. S2 for ASD-specific clinical instrument scores). Due to the nature of the conversational task, only verbal participants took part in the interview, thus limiting severe cases of ASD. Consistent with the clinical literature in child neurodevelopmental health, the cohort is characterized by significant diagnostic comorbidities, with 81% of individuals with ASD also having a comorbid ADHD diagnosis (Fig. 1B). The cohort also included 168 typically developing (TD) youths and 653 participants with other clinical diagnoses, most commonly anxiety and learning disorders (Fig. 1C) without ASD and ADHD. The sample exhibited an overall male-to-female ratio of approximately 2:1 (Fig. 1D), with lower than chance prevalence in females (17% of the ASD group and 23–27% of the ADHD group; Fig. 1E). Note that in Fig. 3E, and in all our analyses, ADHD-Combined presentation is coded as both Inattentive and Hyperactive. To distinguish this coding with two binary variables (Inattentive, Hyperactive) from the three DSM-5 ADHD presentations (Inattentive, Hyperactive, Combined), we will refer to these as diagnostic *status* and *presentation*, respectively.

### Modeling commorbidity and clinical manifestations of ADHD and ASD.

**Figure 2.**
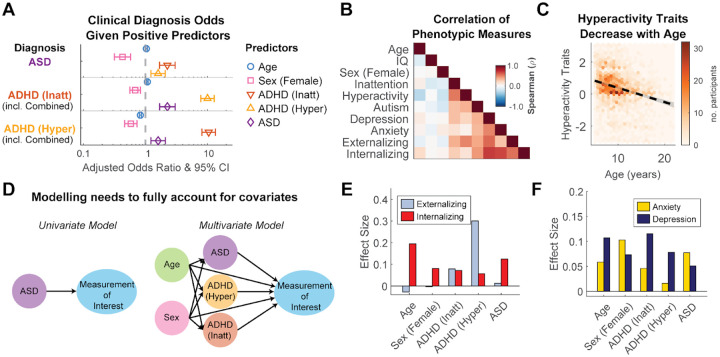
Clinical comorbidity and the necessity of multivariate modeling. **A)** Adjusted odds ratios showing the likelihood of a co-occurring positive diagnosis in individuals with positive predictors (or one-year change in the case of age). Females are less likely than males to receive an ADHD or ASD diagnosis (OR < 1). **B)** Correlation matrix illustrating how clinical instruments aimed at measuring specific neurodevelopmental disorder traits, like ASSQ for autism and SWAN for ADHD, are highly correlated with other measurements like depression (MFQ), anxiety (SCARED), and internalizing and externalizing issues (CBCL), but also with age, IQ, and sex, which makes it difficult to link observed behavior to each of the instrument scales independently. **C)** Two-dimensional histogram showing the negative correlation between age and hyperactivity traits (SWAN scale) (ρ(2194) = −0.28, p<0.001). **D)** Conceptual diagram of the multivariate regression model used to isolate the unique effects of each predictor. A “Univariate Model” ignores covariation, while the “Multivariate Model” controls for shared variance, with Age and Sex as a common source of variance. **E)** Effect sizes from the multivariate model predicting internalizing and externalizing behaviors (CBCL) and **F)** symptoms of anxiety (SCARED) and depression (MFQ). Effect sizes represent the unique contribution of each factor (Sex coded as Female = 1, Male = 0). Full names for clinical instruments (SWAN, ASSQ, MFQ, SCARED, CBCL) are provided in the ‘Clinical Instruments’ section in the Methods. Detailed model statistics for panels A, E, and F are available in Supplementary Materials Sections S1.1 and S1.2.

This community-cohort recapitulates the well-known clinical overlap between ADHD and ASD^12^. A diagnostic status of either ADHD-Inattentive or ADHD-Hyperactive significantly increased the odds of a co-occurring ASD (Adjusted Odds Ratio (OR) = 2.3 and 1.6, respectively) (Fig. 2A). This overlap extends to clinical questionnaires, where trait measures of autism (indexed by the parent ratings of ASSQ^54^), inattention and hyperactivity (indexed by the parent rating of the SWAN-inattention and hyperactive scales^55^, respectively) are not only correlated to each other, but also correlate with other clinical traits, such as anxiety (SCARED^56^), depression (MFQ^57^), as well as age, and sex (Fig. 2B). For instance, the hyperactivity severity decreases with age (ρ(2194) = −0.28, p<0.001, Fig. 2C), consistent with typical development^45^. The same is also reflected in the effect of age on the prevalence of ADHD-Hyperactive status (Fig. 1A, OR = 0.83). Given the well-established effects of development, this “common cause” (age) could induce spurious correlations with ADHD-Hyperactive status for a number of behavioral measures. Similarly, some established associations with ASD may be due to the co-occurring ADHD, or vice-versa.

To isolate the unique contribution of each diagnostic and demographic factor, we employed a multivariate regression model (Fig. 2D), where the clinician-confirmed diagnostic status is coded as binary variables (ASD, ADHD-Inattentive, ADHD-Hyperactive). Effect sizes (ES) for these binary diagnostic status variables will represent Cohen’s d, and Student’s t*√n for continuous variables such as Age and IQ (see Methods). The utility of this approach is clearly illustrated by its application to *externalizing* symptoms, defining outward-directed behaviors such as aggression, rule-breaking, and impulsivity. While in this cohort ASD traits (ASSQ) moderately correlate with externalizing problems (CBCL^58^) (ρ(2119) = 0.38, p<0.001, Fig. 2B), the regression model reveals that this is an artifact of comorbidity. After accounting for all factors simultaneously, externalizing behaviors were predicted by ADHD-Hyperactivity (Effect Size (ES)=0.3), but not ASD (Fig. 2E). On the other hand, we found internalizing problems (inward-directed difficulties like anxiety, depression, and withdrawal) were associated with older ages (ES=0.195), female sex (ES=0.081), and ASD diagnosis and/or ADHD-Inattentive status (ES=0.125 and 0.072, respectively) (Fig. 2E, F). This highlights a shared burden of emotional and behavioral dysregulation in both conditions^59^ and demonstrates the model’s power to parse complex clinical presentations into more specific, disorder-related patterns. Such a model is useful for measures like speech, language, and movement, which are also affected by development^60^, and are the focus of this study.

### Developmental factors dominate over diagnostic effects in structural language

Structural and pragmatic language abilities are typically assessed using standardized psychometric tests, which place heavy demands on non-linguistic abilities like attention and impulse control, potentially confounding results^61^. Here, we used AI-driven tools to extract objective metrics of structural (amount of speech, speech rate, lexical diversity, filler-word usage, etc.) and pragmatic (conversation presence, question-answer coherence, self-referential focus) language abilities from naturalistic conversational responses.

**Figure 3.**
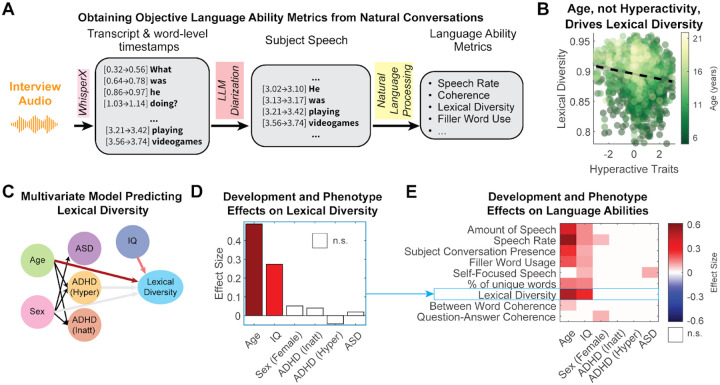
Language abilities are primarily driven by development, not by ADHD or ASD. **A)** The computational pipeline used to extract objective structural and pragmatic language metrics from transcribed audio. **B)** Hyperactive traits (SWAN) and lexical diversity are correlated (ρ(2160)=−0.16, p<0.001). However, the color gradient, representing age, reveals that this association is confounded by development. **C)** Diagram of the multivariate model used to disentangle the effects of diagnosis and developmental covariates on lexical diversity. Arrows are color-coded to the effect size magnitude in the multivariate regression model (panel D). Univariate associations between DSM-5 diagnoses and demographics with lexical diversity (grayed arrows) are instead better explained by age and IQ in the multivariate model. Black arrows indicate effects tested in Fig. 2A. (See Supplementary Materials Section S1.1) **D)** Effect sizes from the multivariate model. **E)** A summary matrix of effect sizes from the multivariate model applied to a range of language metrics. The results show the consistent and strong effects of age and IQ and the general lack of diagnostic effects across all measured aspects of language abilities. Significance cut-off at p < 0.01 (white otherwise; Bonferroni-corrected across the 9 models tested). Detailed multivariate model reports are available in Supplementary Materials Section S1.3.

Our analysis revealed that the structural language abilities measured here were mostly unaffected by either an ADHD or ASD diagnosis (Fig. 2E). Across a range of metrics, our multivariate model, which simultaneously accounts for age, sex, IQ, and diagnosis, consistently confirmed that development (age) was the primary driver of the observed linguistic abilities (Fig. 2E). This confound is illustrated by the apparent relationship between lexical diversity and hyperactivity: while a simple correlation suggested a significant negative association (Fig. 3B, ρ(2160)=−0.16, p<0.001), implying that hyperactivity impairs vocabulary use, our multivariate model (Fig. 3C–D) revealed this variation could be attributed to the stronger effects of age (ES=0.489) and IQ (ES=0.273).

This pattern of a dominant age effect and smaller effects otherwise held across all features we analyzed. For structural language metrics, in addition to age, IQ consistently emerged as a reliable predictor (Fig. 3E). Older participants spoke more (ES=0.398), faster (ES=0.563), and more coherently (ES=0.071). The only significant diagnostic marker was a subtle increase in self-referential speech (i.e., use of first-person pronouns) among participants with ASD (ES=0.1). We also observed secondary demographic effects, with females speaking faster (ES=0.078) and answering more coherently (ES=0.089) than males, and higher IQ and older ages being associated with more frequent use of filler words (ES=0.11 & 0.21, respectively), potentially reflecting the cognitive load of formulating more complex sentences.

### ASD, but not ADHD, is associated with atypical narrative, story comprehension, and perspective taking

**Figure 4.**
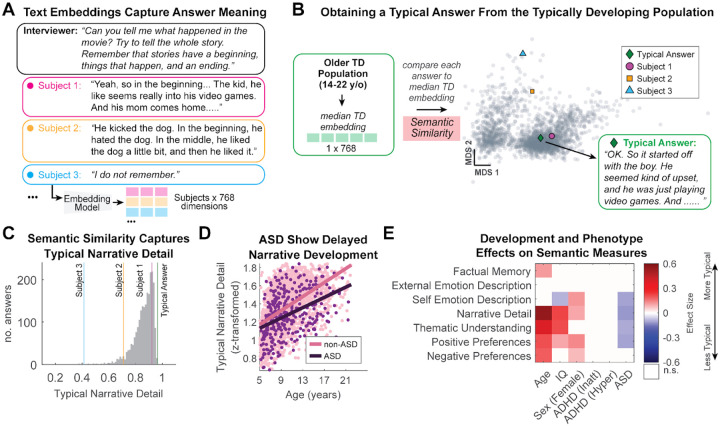
Semantic analysis reveals ASD-specific effects on narrative and social-cognitive abilities. **A)** The computational pipeline for semantic analysis. Participants’ answers to 23 predefined interview questions were parsed and converted into numerical semantic representations (embeddings). **B)** A “typical answer” is obtained from the semantic similarity to the median embedding from the older TD participants (14–22 y/o). **C)** Semantic similarity to the TD prototype is used as a measure of ‘typicality’. For the narrative recall question in panel A, higher similarity reflects a more detailed and typical recount of the narrative. Subject 1 provides a detailed answer (high similarity), whereas Subject 3 does not recall the story (low similarity). **D)** Joint distribution illustrating the positive association between age and the production of more typical narratives, but the diminished abilities in the ASD group compared to the non-ASD group. **E)** Matrix of effect sizes from the multivariate model, assessing the impact of diagnosis and demographic factors on various social-cognitive domains probed by the interview questions (e.g., factual memory, emotional description, and thematic understanding). Only effect sizes with p<0.01, after Bonferroni correction, are shown in color. See Extended Data Table S1 for a full list of questions and Supplementary Section S1.4 for detailed multivariate model reports.

While our analysis revealed that the measured structural and pragmatic language abilities are primarily shaped by development rather than diagnosis, impairments in social communication often stem from difficulties with higher-order functions. These include factual memory, comprehending the central theme of the story, and inferring the mental and emotional states of others, a perspective-taking ability also known as Theory of Mind (ToM). The interview questions were designed to tap into this higher-order functions (see Extended Data Table S1 and Extended Data Table S2). To objectively quantify these functions at scale, we developed a computational pipeline to analyze the semantic content of participants’ interview responses. This approach measured how similar each answer was to that expected from a typical adult (Fig. 4 A–C, assessed here from the older typically developing youths).

The multivariate analysis revealed a distinct pattern of impairment specific to ASD (Fig. 4E). After controlling for the strong associations with age (e.g. Fig. 4D), an ASD diagnosis was uniquely associated with producing less typical and detailed narratives (ES=−0.117), poorer comprehension of the story’s central theme—that both the boy and the puppy in the story were similar because they were missing a leg— (ES=−0.097), and provided more idiosyncratic, or unusual, descriptions of their own emotions (ES=−0.104).

These results provide objective, quantitative evidence for challenges in narrative construction and comprension^62^ (‘Narrative Detail’) and perspective taking^30^ (‘Thematic Understanding’) that are a core feature of autism. In contrast, after accounting for all covariates and multiple-comparison correction, neither the ADHD-Inattentive nor the ADHD-Hyperactive status were significantly associated with any of these social-cognitive measures. Recall that ADHD-Combined was coded as both; therefore, this null result applies to the Combined presentation as well. This suggests that the communication difficulties sometimes attributed to ADHD^63^ do not extend to these functions.

Age remained a powerful predictor across most semantic language use measures (Fig. 4E). Consistent with typical social-cognitive maturation^44^, older participants provided more detailed narratives (ES=0.552), demonstrated a better understanding of the movie’s main message (ES=0.383), and showed more typical positive and negative preferences (ES=0.21 & 0.192). Similarly, higher IQ was associated with more typical narrative abilities (ES=0.223). We also observed a secondary finding related to sex, with females tending to produce more typical narratives (ES=0.103) and less divergent descriptions of their emotions (ES=0.119) and positive preferences (ES=0.137) compared to male participants (Fig. 4E). In none of our analyses did we see a marked interaction of these effects with diagnostic status. One exception was the age effect ASD on ‘Narrative Detail’ which tended to increase with age (Fig. 4D; see follow-up analysis under Statistical Methods).

### Autism Spectrum Disorder has a distinct vocal profile

**Figure 5.**
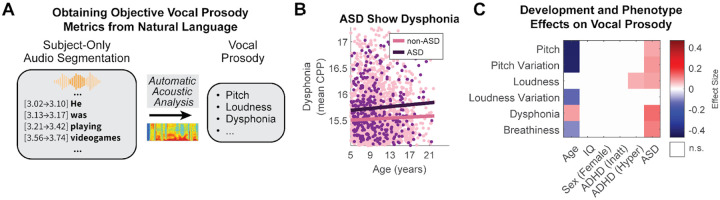
Automatic vocal prosody metrics reveal an ASD-specific acoustic profile. **A)** Automatic extraction of vocal prosody features involved transcribing and diarizing speech, thus isolating the children’s speech from that of the adult interviewer, and extracting audio-based features using an open-source toolbox (OpenWillis^67^). **B)** ASD participants show increased dysphonia compared to non-ASD participants, which may be related to an “unusual” voice in ASD. **C)** Effect Size matrix illustrating the effects of development and ASD on vocal prosody. Effect Sizes in this matrix are estimated again using a multivariate regression model, with p<0.01, after Bonferroni correction. See Supplementary Materials Section S1.5 for detailed multivariate model reports.

The acoustic patterns of pitch, intensity, and rhythm are critical for conveying the intent of an utterance, and atypical prosody is a widely reported clinical feature of ASD^32^. To this end, we employed an automated pipeline to extract a comprehensive set of acoustic features from the segmented speech of each child, including measures of pitch, intensity (loudness), and voice quality (Fig. 5A).

Our multivariate analysis revealed a distinct acoustic signature specific to ASD. After controlling for age, sex, and IQ, an ASD diagnosis was significantly associated with increased mean pitch (ES=0.079), greater pitch variability (ES=0.094), and higher vocal loudness (ES=0.083) (Fig. 5 C). Furthermore, we identified significant markers related to voice quality: participants with ASD exhibited significantly higher dysphonia severity (ES=0.133, Fig. 5B), as measured by cepstral peak prominence (CPP)^64^, and increased breathiness (ES=0.117), measured by the glottal-to-noise excitation ratio^65^. These quantitative findings align with clinical descriptions of an “unusual” voice in ASD^66^. Notably, this vocal profile was specific to ASD. Consistent with our findings for language content, narratives, and perspective taking, neither an ADHD-Inattentive nor an ADHD-Hyperactive status (which includes ADHD-Combined) was associated with any of the measured vocal prosody features. Developmental effects were also prominent, with age being a significant predictor of reduced pitch (ES=−0.419), pitch and loudness variability (ES=−0.415 & −0.14), and to a lesser degree breathiness (ES=−0.104), and dysphonia (ES=0.086). Together, these results identify an objective vocal signature for ASD that is independent of ADHD status and comorbidity and follows a clear developmental course.

### Increased motor activity is uniquely associated with ADHD-Hyperactive diagnostic status

**Figure 6.**
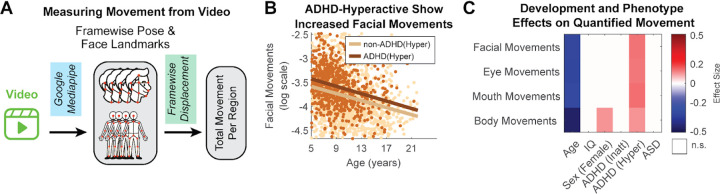
Associations of demographics and phenotype with body and face movements. **A)** We use Google’s Mediapipe computer vision models to obtain framewise locations for anatomical and facial landmarks. We then calculate the average framewise displacement for the different regions of interest. **B)** ADHD-Hyperactive youth show increased facial movements across the whole developmental spectrum, compared to non-ADHD Hyperactive. **C)** Effect Size matrix illustrating the effects of development and an ADHD-Hyperactive status on all measures of face and body movements. Effect Sizes in this matrix are estimated using multivariate regression, with p<0.01, after Bonferroni correction. See Supplementary Materials Section S1.6 for detailed multivariate model reports.

Motor behaviors are relevant diagnostic features of ASD and ADHD, with hyperactivity being a key diagnostic criterion in ADHD, and stereotypical and repetitive motor behaviors being among the defining criteria for ASD. Yet motor behaviors are rarely quantified objectively in naturalistic settings. We applied computer vision models to the interview videos to extract objective metrics of upper-body and facial movement. Using pose and facial landmark estimation models, we computed the average frame-to-frame displacement for several regions of interest, including the upper body, head, eyes, and face (Fig. 6A).

Consistent with a typical developmental trajectory, age was a strong negative predictor of movement across all measures (ES ranging from −0.31 to −0.46). After accounting for these developmental effects, we identified a specific motor signature. ADHD-Hyperactive status was uniquely and robustly associated with increased movement across all measured body and facial regions (ES=0.10–0.14) (Fig. 6B–C). Conversely, neither ASD nor ADHD-Inattentive were linked to any significant changes in motor activity. We also observed a significant association between female sex and increased overall body movement (ES=0.105). These results demonstrate that while increased motor activity is a powerful, objective marker, it is specific to the ADHD-Hyperactive diagnosis and does not generalize to ASD or the Inattentive diagnosis in our population.

## Discussion

This study used digital phenotyping to objectively analyze communication and motor behavior across a wide developmental range in youths with ADHD and/or ASD. We used relatively short videos from a scripted interview to identify objective markers of these domains using artificial intelligence. By using a large, community-recruited cohort, this study captured the variability seen in real-world clinical settings.

The dominant driver of variance in our measures of language, speech, and motor behavior was age. Language deficits in ADHD were fully explained by the confounding effect of age and co-occurring ASD. Conversely, ASD showed structurally intact language but significant impairments in narrative ability and perspective-taking, coupled with a unique vocal profile.

Previous research, often using standardized assessments, has often linked ADHD to language disturbances affecting both structural and pragmatic, or social, aspects of communication^63,68^. This view is supported by community-based studies using standardized language assessments, which have found that children with ADHD are nearly three times more likely to have co-occurring language problems than their typically developing peers, with prevalence rates of impairment estimated between 40–45%^69,70^. Our findings, derived from objective quantification of spontaneous conversation, challenge this view. After accounting for developmental factors such as age and IQ, we did not detect a consistent association between an ADHD diagnosis and structural or pragmatic language abilities, aligning with findings from other naturalistic studies^61^. In other words, the changing prevalence of ADHD with age and its association with IQ may have confounded previous language studies that do not take these factors into account. Additionally, the discrepancy between our findings and studies reporting high rates of language impairment may be explained by methodological differences. Standardized psycholinguistic tests often place significant demands on non-linguistic abilities such as sustained attention, impulse control, and working memory capacity^71^. Poor performance on these assessments by children with ADHD may therefore reflect these core executive function deficits rather than a primary limitation in language abilities^61^.

The finding that apparent language deficits are better explained by executive dysfunction extends from basic structural language abilities to higher-order social cognition. Our investigation of narrative ability, story comprehension, and perspective-taking (ToM) found that ADHD was similarly unassociated with any of these domains, in opposition to some previous studies^72^. These social-cognitive functions were, like language abilities, strongly moderated by age and IQ, but were uniquely and significantly impaired in participants with ASD. This suggests that the pragmatic communication challenges in ADHD are, in a similar fashion, more likely linked to underlying executive function deficits, such as poor inhibition and planning, which impact the organization and delivery of speech, rather than a fundamental deficit in understanding language or the mental states of others^73,74^. Our results reinforce the view that impaired perspective taking (ToM) is a critical differentiator between ASD and ADHD^75^ and suggest its presence may serve as a strong indicator of comorbid ASD in individuals with ADHD.

In contrast to these findings for ADHD, our results for ASD provide quantitative support for the classic conceptualization of the disorder, defined by the core challenges in social communication^5^. In this study, participants with ASD performed similarly to their peers on structural language measures like lexical diversity and coherence, with their language abilities following a typical developmental trajectory. The one discernible feature at this level was a greater tendency for self-referential speech, marked by an increased use of first-person pronouns. This subtle linguistic pattern may serve as an objective marker of the self-focused perspective often described in autism, and echoes findings of pronoun use abnormalities that suggest an asynchrony between linguistic and social development^76^.

The most prominent communication challenges in our ASD cohort emerged clearly in these higher-order domains of narrative and social cognition. Our semantic analysis revealed that participants with ASD produced less “typical” narratives, suggesting a divergence in identifying and recounting the most socially salient aspects of the story. They also were less likely to grasp the film’s central theme, pointing to a challenge in moving beyond literal events to understand the overarching message or ‘gist’ of the story^62^. Most notably, their idiosyncratic emotion descriptions provide quantitative evidence of the ToM-related challenges that are characteristic of the condition^77^. These results, obtained at an unprecedented scale and in a naturalistic, conversational context, both validate and extend foundational work that used computational natural language processing to objectively measure narrative skills in autism. Previous studies, which primarily employed Latent Semantic Analysis, had already demonstrated that individuals with ASD show diminished narrative quality in demanding recall tasks^78^ and in response to emotionally evocative scenes^79^. Our findings confirm that these core deficits are promising candidate digital markers for ASD.

This study further provides a quantitative basis for the long-observed atypical prosody in ASD, which, despite being clinically apparent, has been difficult to define with objective, replicable metrics^35^. Our large-scale analysis robustly confirmed the findings of increased pitch and pitch variability^34^, but also clarified these prior inconsistencies by demonstrating a significant and consistent increase in vocal intensity (loudness) in our ASD cohort. Furthermore, we identified a distinct acoustic profile characterized by increased dysphonia and breathiness as markers of ASD, providing objective validation for long-standing clinical descriptions of autistic voices as “hoarse” or “creaky”^66^. Most importantly, these associations were absent in ADHD, revealing yet another distinct marker in both disorders.

Complementing the analysis of communication, our objective quantification of motor behavior revealed a particular signature that further differentiates the clinical presentations of ADHD and ASD. Motor difficulties are a well-documented feature of ADHD, but are typically measured with subjective rating scales or specialized sensors like actigraphy^43^. Our study demonstrates the feasibility of using unobtrusive, scalable computer vision methods to quantify motor activity in a naturalistic conversational setting, a technique with significant potential for both clinical and in-home monitoring^80^. We found that, consistent with typical development, overall movement decreased with age. After accounting for this maturational trend, increased movement of the head, body, and face was a specific marker of an ADHD-Hyperactive diagnostic status.

Conversely, our analysis did not find an association between increased movement and diagnoses of either ADHD-Inattentive or ASD. This null finding reinforces the specificity of our measure to the hyperactive-impulsive domain of ADHD. The absence of a detectable general motor signature for ASD in the present work, however, warrants careful interpretation. Motor challenges are increasingly recognized as a core feature of ASD^42^. It is likely that the global displacement metrics used in our study, while effective at capturing the overt restlessness of hyperactivity, are not sensitive to the repetitive motor patterns characteristic of autism^41^. The moderate levels of autism in our sample may also mean that stereotypical motor mannerisms were less apparent in this context. Future work could employ more granular computer vision techniques, such as those that classify specific gesture types or postural patterns^81–85^, to better characterize the nuanced motor profile of ASD^41,86^. Nevertheless, our findings establish objective, video-derived hyperactivity as a robust and specific digital biomarker for the ADHD-Hyperactive diagnostic status.

Finally, our results consistently highlight the influence of age and sex across all measured behavioral domains. Age was a powerful predictor of maturation, driving changes in everything from fundamental motor control and vocal pitch to the complexity of socio-cognitive and linguistic abilities. Similarly, distinct sex-specific profiles emerged in vocal patterns, language, and body movement. Developmental trajectories and demographic factors are not merely noise to be controlled for, but essential elements of the behavioral phenotype. Any search for valid, disorder-specific digital biomarkers must therefore be built upon developmental models that can successfully disentangle the effects of pathology from the natural course of maturation and sex-based differences.

Beyond these demographic and developmental factors, our models also included general cognitive ability by using Full-Scale IQ as a covariate. We acknowledge that IQ is not a pure independent measure of aptitude but partially an outcome that is affected by the disorder itself^87^. Statistically, it can behave similarly to sex, which is associated with the disorder due to varying prevalence. In the present cohort, there is a mean drop in IQ of approximately 10 points from typically developing kids with a positive diagnosis of ASD or ADHD (Table 1). However, IQ also varies substantially within each diagnostic status, with a variance that is no different than in typically developing youths (Table 1). In particular, it is widely recognized that cognitive ability can vary widely within ASD^48,49^. It is therefore expected to serve as an independent predictor, for instance, of language abilities in ASD^32,88^. Nevertheless, to ensure the robustness of our core findings, we repeated our primary analyses without including IQ as a covariate. The significant associations between diagnostic status and our key behavioral markers for ASD and ADHD remained consistent (see Extended Data Fig. S3). Similarly, the results did not change if using verbal IQ scores as a covariate instead.

Current clinical practice includes the use of established clinical instruments that measure behavioral presentations. As one may expect, our measures of movement, language, and speech correlate strongly with these established metrics (see Extended Data Fig. S4). However, it appears that our automated features are at times more specific or more sensitive to the ADHD and ASD presentations. However, we want to clarify that we are not suggesting these automated behavioral features should replace current diagnostic tools. Traditional diagnostic clinical instruments appear to be better matched to the clinical categories as currently defined in the DSM-5 (see Extended Data Fig. S5).

Several limitations should be noted. Our sample primarily consisted of mild to moderate ASD, which might explain the absence of behavioral stereotypes more commonly observed in more severe cases. Although our models accounted for the significant overlap between ADHD and ASD, other co-occurring conditions could have influenced the results. However, we ruled out the impact of child anxiety on our behavioral measures by repeating all analyses while modeling for co-occurring positive anxiety diagnoses, and the associations with ADHD or ASD remained consistent (Extended Data Fig. S6). To unambiguously assign variance to the Hyperactive and Inattentive status, we chose not to code for Combined presentation, as in some previous works^89–91^, however, when coding in the regression analysis instead for Inattentive and Combined presentation, the results are identical (Extended Data Fig S7). Furthermore, as a cross-sectional study, we could identify developmental trends across different ages but could not track changes within a single child over time or establish causality. Future work should validate our brief, video-based measures against long-term behavioral data, such as from wearable sensors, to ensure they accurately capture a child’s typical behavior. Applying these computational tools in other critical settings, including parent-child interactions and standardized assessments like the ADOS, will also be essential to confirm their broader clinical utility.

In summary, our multivariate analyses, accounting for developmental effects, identified distinct behavioral tendencies that differentiate ADHD and ASD at the group level. ADHD, particularly the hyperactive-impulsive and combined presentations, were characterized by increased motor activity, while language and narrative difficulties did not emerge as robust markers once development and comorbidity were considered. Conversely, ASD exhibited a unique social-communication signature, with mostly typical structural language but notable impairments in narrative skills and perspective taking, along with a distinctive vocal profile characterized by higher pitch, greater intensity, and altered voice quality. These results demonstrate that digital phenotyping can provide objective, scalable, and developmentally sensitive markers to help differentiate overlapping neurodevelopmental disorders.

## Methods

### Clinical Population

This study used video-recorded conversations with 2,341 children (aged 5–22, M=10.1, SD = 3.39 years) as part of the Healthy Brain Network (HBN)^50^. The cohort included 1,455 participants with clinician-confirmed ADHD: 653 with inattentive presentation, 93 with hyperactive-impulsive presentation, and 625 with combined presentation. An additional 84 showed ADHD traits without meeting diagnostic criteria for a specific ADHD presentation (DSM-5 ADHD-other/unspecified). The sample also comprised 349 individuals with mild to moderate ASD, with 81% of ASD cases also having a comorbid ADHD diagnosis of some type. The cohort included 168 typically developing individuals and 653 with other clinical diagnoses, mostly anxiety and learning disorders. The overall male-to-female ratio was 1.98:1 (1556/785). Detailed demographic and clinical characteristics per diagnosis are available in Table 1.

Of the 2,341 participants, 1143 (48.8%) were white/caucassian, 311 (13.3%) black/african american, 226 hispanic (9.7%), 55 (2.3%) asian, and 42 (1.8%) identified as other races. 382 (16.3%) identified as two or more races. 182 (7.8%) participants did not specify, or their race was unknown.

### Behavioral Task and Data Acquisition

Participants were recorded during a semi-structured interview conducted in a controlled laboratory setting. As part of the HBN protocol, participants watched “The Present,” a short, emotive animated film, and were then prompted by a clinician to narrate the story and answer perspective-taking questions in the form of a scripted interview. High-definition video of the participant’s face and upper body was captured using a Canon XC15 camcorder, while high-fidelity audio was recorded simultaneously with a Røde NT1 microphone. This scripted procedure provided the raw data for subsequent automated analysis of motor, prosodic, and linguistic features.

### Diagnostic Procedure

The HBN is a large-scale biobank that utilizes a community-referred recruitment model to capture the wide heterogeneity inherent in developmental psychopathology. As such, the HBN sample is enriched for individuals meeting DSM-5 criteria, though cases are not typically severe. Diagnoses within the HBN are determined by licensed clinicians at the Child Mind Institute based on a consensus model that integrates multiple sources of information. The primary diagnostic instrument is the semi-structured Kiddie Schedule for Affective Disorders and Schizophrenia (K-SADS)^92^ for DSM-5, which is administered to both participants and their parents. The final consensus diagnosis also incorporates behavioral observations during testing, parental and self-reported clinical questionnaires, and the participant’s developmental and educational history. For ASD, the primary evaluation relies on the KSADS autism module and questionnaires (e.g., SRS-2, SCQ, ASSQ), with only a limited subset of participants receiving supplemental “gold-standard” assessments like the ADOS or ADI-R.

For our analysis, clinician-confirmed diagnoses were modeled to assess the impact of specific disorders. ADHD diagnoses were categorized as either primarily inattentive or hyperactive/impulsive diagnoses. Participants with a clinician-confirmed diagnosis of ADHD-Combined presentation were considered positive for both hyperactive and inattentive status. ASD diagnoses were modeled as a single binary variable (1 for positive, 0 for negative). The vast majority of these individuals (>95%) were mild to moderate (only a few requiring maximum support), consistent with the HBN’s inclusion criteria, which require participants to be verbal (see Extended Data Fig. S2 for distribution of autism clinical instrument scores). The control group, designated as the Typically Developing Population, consisted of individuals with a confirmed negative diagnostic clinician decision in all DSM-5 categories.

### Clinical Instruments

Participants in the HBN are deeply phenotyped using a comprehensive battery of clinical instruments. For this study, we defined ADHD traits using the Strengths and Weaknesses of ADHD Symptoms and Normal Behavior Scale (SWAN)^55^, and ASD traits using the Autism Spectrum Screening Questionnaire (ASSQ)^54^. These scales were selected as they showed the strongest unique association with their respective clinician-confirmed diagnoses compared to other available scales (see Extended Data Fig. S5). Other clinical instruments used in this study include the Mood and Feelings Questionnaire (MFQ, depressive traits)^57^, Screen for Child Anxiety Related Disorders (SCARED-P, anxiety traits)^56^, and the Child Behavior Checklist (CBCL)^58^, with two factors: internalizing and externalizing traits. A full description of age, sex, IQ, and clinical instrument scale scores for each diagnostic group is provided in Table 1.

### IQ score

Cognitive ability was assessed using a composite IQ score. Full-Scale IQ scores were primarily measured using the Wechsler Intelligence Scale for Children (WISC-V)^51^ (N=1984, mean=99.2, SD=16.7). Exceptions included early participants who were administered the Wechsler Abbreviated Scale of Intelligence (WASI)^52^ (N=36, mean=97.8, SD=14.7), and children under age 6 or with known IQ below 70, for whom the Kaufman Brief Intelligence Test (KBIT)^53^ was used (N=166, mean=100.4, SD=15.8). Full-Scale IQ Composite scores were available for 2186 out of the 2341 participants (93.3%). Similarly, verbal IQ scores were obtained by combining the WISC-VCI (N=1986, mean=103.6 SD=16.6), WASI-VCI (N=36, mean=99.3 SD=14.4), and KBIT-verbal (N=166, mean=100.6 SD=15.9) scores. Verbal IQ scores were available for 2188/2341 participants (93.5%). Main analysis and results were computed using Full-Scale IQ as covariate. However, main associations between diagnoses and measured behavior were independent of including IQ as covariate or not (Extended Data Fig. S3). Similarly, the results did not change significantly if using Verbal IQ scores instead.

### Transcription, Diarization, & Question-Answer extraction

The audio recordings were first transcribed into text using the WhisperX model (v.3.3.0, Whisper model *large-v2*)^93^ in a local workstation, which generated a raw transcript with timestamps for each word. Following transcription, speaker diarization was performed to separate the raw text into words spoken by the interviewer and the participant. This was achieved using a custom prompt with Google’s large language model (*gemini-2.0-flash*). The diarized transcript was then used for a final extraction step, where a separate LLM prompt isolated the specific questions from the protocol and the full corresponding answers provided by the participant. A list of the scripted questions, as well as the “typical answer”, i.e. the answer semantically closest to the median embedding of the older typical population (14–22 y/o), for each question, the typical answer is given in Extended Data Table S1 (see ‘Semantic Analysis’ method section for details). The complete prompts for LLM-based diarization and question-answer extraction are available in the Supplementary Materials Sections S2 and S3.

### Description of Behavioral Variables

Various open-source tools were used to obtain objective measures of language, speech prosody, semantic content, and movement from the interview data. A summary of the measures is provided in Extended Data Table S2.

#### Language and Speech Prosody Measures.

We extracted a set of acoustic and linguistic features using the Openwillis toolkit (v.3.0.5)^67^. Openwillis is a Python wrapper developed by Brooklyn Health that automates feature extraction by integrating established open-source speech and natural language processing tools for digital phenotyping, and can operate locally.

#### Semantic Measures.

To characterize the semantic content of participants’ answers, we utilized Google’s ‘Gecko’ text embedding model (*text-embedding-004*) to generate 768 dimensional numerical representations of each response^94^. First, we established a normative semantic baseline by calculating the median embedding for each of the 23 questions across all answers from the old participants in the typically developing (TD) population (14–22 y/o). Then, for each participant’s answer, we computed its cosine similarity to the corresponding TD median embedding. This yielded a score representing how semantically ‘typical’ the response was. Participants with missing answers were assigned a missing score for that question. The interpretation of this score depends on the question’s content; for narrative-recount questions, higher similarity reflects greater “typical narrative detail,” whereas for questions about emotion, lower similarity may indicate more nuanced or idiosyncratic descriptions. Finally, these 23 individual typicality scores were averaged into conceptually meaningful domains based on the function of the questions. These domains included narrative detail, factual memory, thematic and emotional understanding, and perspective taking (e.g., describing one’s own or a character’s emotions, or the similarity between the dog and the kid and how this relates to the kid’s behavior). For example, the ‘self-emotion description’ score was the average semantic similarity score across answers to questions 14, 17, 20, and 23 (see Extended Data Table S1 for details on these questions and examples of the ‘Typical Answers’). The Fisher-Z transform (inverse hyperbolic tangent, artanh) was applied to the semantic (cosine) similarity measures prior to regression analysis to approximate normal distributions.

#### Movement measures.

We extracted anatomical and facial landmarks from video recordings at the native 30Hz sampling rate of the video recordings using Google’s Mediapipe Holistic model (v.0.10.11) in a local machine. The raw 3D landmark coordinates were filtered to remove low-confidence data based on two criteria applied over a sliding window. First, segments with more than 10% missing values in a 2-second window were excluded. Second, segments exhibiting excessive jitter, defined as a standard deviation of the x-coordinate exceeding empirically-defined thresholds (0.1 for face; 0.01 for pose and landmarks) in 10-frame windows, were also excluded. A landmark point was considered valid for analysis only if it passed both of these checks. To ensure comparability across participants, the filtered data were normalized. Facial landmarks were aligned to a canonical model using an affine transformation^95^ to correct for head rotation, translation, and scale. Body landmarks were normalized by the frame-by-frame inter-shoulder distance to render movements invariant to body size and participant’s distance from the camera. Finally, movement was quantified as the frame-to-frame 3 D Euclidean displacement for each landmark. These displacement values were then averaged within predefined anatomical regions (e.g., face, mouth, eyes, and body) and across all valid frames to yield a single mean movement score per region for each participant. Log of the average movement was used to model the effects of diagnosis on such measures.

### Analytical Approach & Statistical Modeling

To test our primary research questions, we modeled the relationship between the measurements of interest and clinical diagnoses using multivariate linear regression. The model isolated the unique contribution of ASD and ADHD diagnostic status while controlling for key developmental and cognitive confounders. For each behavioral metric, the statistical model took the following form:

(1) Metric~Age+IQ+Sex+ADHD-Inatt+ADHD-Hyper+ASD

with one model for each dependent variable (e.g., speech rate, lexical diversity), and the predictors were age (in years), a composite IQ score (Full-Scale), Sex (coded binary female=1, male=0), and binary variables for clinician-confirmed diagnoses of ASD, ADHD-Inattentive, and ADHD-Hyperactive (ADHD-Combined presentation was modeled as positive in both). Every participant counts as n=1, with a set of numbers describing them: Age, IQ, ASD, ADHD-Hyper, ADHD-Inatt. For instance, an 8 year old boy with an IQ of 90 who presents with ASD and ADHD-Combined is coded as: 8, 90, 0, 1, 1, 1. Models were fitted using the bisquare ‘robust’ linear fitting option in MATLAB (2024b). To quantify and compare the effect of each predictor, we estimated its effect size from the t-statistic and error degrees of freedom DFE:

(2) $$\text{ES}=\text{t-stat}/\text{sqrt}\left(\text{DFE}\right)$$

This normalization allows for the comparison of effects across different variables, even when the number of observations varies due to missing data for a specific behavioral metric. This measure is the traditional Cohen’s d, in the case of binary variables (Sex and Disorders), and a sensible measure of effect size (mean over standard deviation of the beta coefficient) in the case of regression with continuous variables (Age and IQ). Statistical tables for each model of the behavioral outcome measures, including all beta coefficients, standard errors, t-statistics, effect sizes, and p-values, are provided in Supplementary Materials Sections S1.3–S1.6.

For continuous outcome measures such as the trait measures (SCARED, MFQ, CBCL-Internalizing, CBCL-Externalizing), we use the same model as with the behavioral outcome measures above (Fig. 2E–F, Supplement Materials Section S1.2).

For binary outcome measures (clinical diagnosis of ASD, ADHD-inattentive, and ADHD-hyperactive), we use a logistic binomial model and report odds ratios (OR) as the exponents of the beta coefficients (Fig. 2A and black arrows in Fig. 3C, Supplementary Materials Section S1.1).


(3) $$\text{OR}=\text{exp}\left(\beta \right).$$

Note that the linear model (1) does not include interaction terms. For age in particular, this means that the effects of the disorders are modeled as a constant gap between a positive and a negative diagnosis. However, for some behavioral features, the gap appears to increase with age (e.g., Fig. 4D, 5B). Such an overall gap can be detected by model (1) even if it increases with age. Given the reduced statistical power of larger models, we have therefore avoided interaction terms. Nevertheless, we performed a post-hoc analysis including an Age*Disorder interaction whenever there was a significant finding of age and ASD, ADHD-Hyperactivity, or ADHD-Inattention diagnostic status. This follow-up analysis suggests that there is an increasing gap with age for narrative detail in ASD (Fig. 4D, p=0.014 uncorrected), and for body movement in ADHD-Hyperactivity (Fig. 6C, p=0.044 uncorrected). Conversely, differences appear to diminish with age for pitch, pitch variation, and breathiness in ASD (Fig. 5C, p=0.004, 0.022 & 0.029, uncorrected).

## Supplementary Material

1

## Figures and Tables

**Table 1.** Demographic, clinical, and interview characteristics

Characteristic	ADHD-Inattentive (incl. Combined) (N=1278)	ADHD-Hyperactive (incl. Combined) (N=718)	ASD (N=349)	Typically Developing (N=168)	Full Sample (N=2341)
**Age (in years), mean (SD)**	10.1 (3.22)	9.1 (2.91)	10.3 (3.69)	9.4 (3.23)	10.1 (3.39)
**Sex, N (%)**					
Female	346 (27)	162 (23)	60 (17)	74 (44)	785 (34)
Male	932 (73)	556 (77)	289 (83)		1556 (66)
**Co-morbidities, N (%)**					
ADHD-Inattentive	N/A	625 (87)	264 (76)	0 (0)	1278 (55)
ADHD-Hyperactive	625 (49)	N/A	166 (48)	0 (0)	718 (31)
Autism Spectrum Disorder	264 (21)	166 (23)	N/A	0 (0)	349 (15)
**Clinical Instruments, mean (SD)**					
Inattention (SWAN)	1.2 (0.9)	1.1 (0.9)	1.1 (1.0)	−0.3 (1.0)	0.7 (1.1)
Hyperactivity (SWAN)					0.4 (1.1)
Autism (ASSQ)	8.5 (9.2)	10.2 (10.1)	17.5 (10.9)	2.3 (3.1)	7.1 (8.6)
Depression (MFQ)	10.0 (8.8)	9.8 (8.0)	10.3 (8.8)	4.7 (4.9)	9.0 (8.6)
Anxiety (SCARED)	14.7 (12.1)	14.6 (12.0)	16.5 (12.9)	9.4 (8.0)	14.2 (11.8)
Externalizing (CBCL)	12.6 (9.8)	15.5 (9.8)	12.4 (9.5)	5.6 (5.7)	10.9 (9.5)
Internalizing (CBCL)	10.5 (8.3)	10.5 (8.0)	12.2 (8.3)	5.3 (5.0)	9.8 (8.2)
**IQ, mean (SD)** *					
Full-Scale	98.5 (16.3)	100.3 (16.2)	96.7 (18.9)	106.5 (14.3)	99.4 (16.6)
Verbal	103.0 (16.3)	104.1 (16.2)	100.8 (19.4)	108.8 (14.3)	103.2 (16.5)
**Interview length (minutes), mean (SD)**	4.5 (1.51)	4.5 (1.47)	4.7 (1.62)	4.5 (1.95)	4.5 (1.54)

* IQ was measured using WISC-V^51^, with exceptions for early participants using WASI^52^, and children under 6 or with IQ below 70 using KBIT^53^. See ‘IQ Score’ section in Methods for details.

## Data Availability

Phenotypical, diagnostic, and interview data are available upon request and with the establishment of a Data Use Agreement with the Child Mind Institute at https://fcon_1000.projects.nitrc.org/indi/cmi_healthy_brain_network/.

## Code Availability

The full code pipeline used for speech transcription and diarization, as well as to extract language, vocal prosody, semantic embeddings, and body and face landmarks, is publicly available at https://github.com/asortubay/AI_behavioral_analysis.
