# Supplementary material for: Overlap and Differences of Autism and ADHD: Digital Phenotyping of Movement and Communication During Development
Source: bioRxiv. 2025 Oct 20:2025.10.20.682864. Preprint. [Version 1] doi: 10.1101/2025.10.20.682864 (PMC12633450; doi:10.1101/2025.10.20.682864)
Supplement: 1 [file NIHPP2025.10.20.682864V1-supplement-1.pdf]

bioRxiv preprint doi: https://doi.org/10.1101/2025.10.20.682864; this version posted October 20, 2025. The copyright holder for this preprint (which was not certified by peer review) is the author/funder, who has granted bioRxiv a license to display the preprint in perpetuity. It is made available under aCC-BY 4.0 International license.

Extended Data

Extended Data Table S1. Interview Questions & Typical Answer

Semantic Measure		Question	Typical Answer, Typically Developing Children
—	1	"I hope you enjoyed the last movie. Have you seen it before?"	"Yes. Yeah."
Narrative Detail	2	"Can you tell me what happened in the movie? Try to tell the whole story. Remember that stories have a beginning, things that happen, and an ending."	"OK. So it started off with the boy. He seemed kind of upset, and he was just playing video games. And it was kind of dark in their house and stuff. And his mom comes home, and she like turns on the lights and everything and she gives him, she gives him the gift in the box so he stops playing and then he opens the box and he like sees a puppy and then he kind of gets excited and then he sees that the puppy doesn't have a leg, like is missing one leg. So then he's like not interested and he kind of just puts it off to the side but the dog doesn't really know what's going on. It's just like excited to be with someone like an owner or something. And then he goes back to playing video games, and the dog is kind of just searching around the house and finds the ball. And you can tell that the dog's kind of having trouble walking and playing with it and stuff. And so then he just kind of watches the owner. He kind of watches as the dog's kind of struggling. but he's playing with the ball and it's kind of making him kind of happy watching him just like play around and he's like still happy. And then he eventually turns off his video game and then it ends when they go outside to play. And you realize that he has some, like he, his leg also is missing."
—	3	"Do you remember anything else from the story?"	"Um... No. Okay."
Positive Preferences	4	"What are some of the things you liked about the movie?"	"I liked the dog. And I also liked how he liked him at the end and started playing with him. Yeah, that's nice."
Negative Preferences	5	"Is there something you didn't like about the movie?"	"There wasn't a whole lot that I didn't like. I mean, like, honestly, I didn't like the fact that he was being mean in the beginning, kicking the dog."
Factual Memory	6	"Who gave the boy a box?"	"The mom."
Factual Memory	7	"What was in the box?"	"A dog."
Factual Memory	8	"What was the boy doing before he got the box?"	"Playing video games."
Factual Memory	9	"What was the puppy playing with?"	"A ball, red ball."
Thematic Understanding	10	"How are the puppy and the boy the same?"	"They both are missing a leg."
—	11	"In the movie, who is missing a leg? The boy, the puppy, or no one?"	"Both."
	Kids rewatch a short clip from "The Present" (00:53-01:02)		
External Emotion Description	12	"How do you think the puppy was feeling?"	"Happy. Really happy."
External Emotion Description	13	"How do you think the boy was feeling?"	"Happy. A little excited."
Self Emotion Description	14	"And how did you feel while you were watching that part?"	"Happy."
	Kids rewatch a short clip from "The Present" (01:03-01:09)		
External Emotion Description	15	"How do you think the puppy was feeling?"	"At first he was okay and then he was probably upset."
External Emotion Description	16	"How do you think the boy was feeling?"	"Disappointed, a little angry, maybe."
Self Emotion Description	17	"And how did you feel while you were watching that part?"	"A little bit upset, because it was pretty sad and all that."
	Kids rewatch a short clip from "The Present" (01:14-01:23)		
External Emotion Description	18	"How do you think the puppy was feeling?"	"Sad. Like he tried again to like play and all that and then he just kicked him over to get lost."
External Emotion Description	19	"How do you think the boy was feeling?"	"Angry. Still mad."
Self Emotion Description	20	"And how did you feel while you were watching that part?"	"A little bit sadder because he was actually kind of unleashing his emotions at the puppy.."
	Kids rewatch a short clip from "The Present" (03:03-03:21)		
External Emotion Description	21	"How do you think the puppy was feeling?"	"Happy."
External Emotion Description	22	"How do you think the boy was feeling?"	"Happy, too."
Self Emotion Description	23	"And how did you feel while you were watching that part?"	"Good. Happy."

Extended Data Table S2. Description of Behavioral Variables.

bioRxiv preprint doi: https://doi.org/10.1101/2025.10.20.682864; this version posted October 20, 2025. The copyright holder for this preprint (which was not certified by peer review) is the author/funder, who has granted bioRxiv a license to display the preprint in perpetuity. It is made available under aCC-BY 4.0 International license.

Group	Measured Aspect	Variable	Measurement
Language Ability Measures	Structural Language	*Amount of Speech*	Total subject speech length in words
		Speech Rate	Subject speech rate, in words per minute
		Filler Word Usage	Number of filler words ('um', 'uh', 'er', 'ah', 'like', 'you know', 'so', 'okay', 'right') per minute of subject speech
		% of unique words	Percentage of unique words in total subject speech
		Lexical Diversity	Average Moving Average Type Token Ratio with a window size of 10
		Between Word Coherence	Average measure of semantic similarity of each word to the preceding word using LLMs
	Pragmatic Language	*Subject Conversation Presence*	Subject speech percentage from total conversation speech
		Self-Focused Speech	Percentage of pronouns in the utterance that are first-person singular pronouns
		Question-Answer Coherence	Average semantic similarity of current speaker turn to previous turn of the other speaker
Semantic Measures (Socio-Cognitive Abilities)	**Memory**	*Factual Memory*	Answer Typicality when asked about facts in the movie
	**Social Judgment & Salience**	*External Emotion Description*	Answer Typicality when asked about dog/child emotions in the movie
	**Theory of Mind**	*Self Emotion Description*	Answer Typicality when asked about self emotions in the movie
	**Narrative Production, Comprehension & Theory of Mind**	*Narrative Detail*	Answer Typicality when asked to give a detailed recall of the movie
	**Theory of Mind**	*Thematic Understanding*	Answer Typicality when asked about the similarities of the dog and kid, and their shared experience
	**Social Judgment & Salience**	*Positive Preferences*	Answer Typicality when asked about what they liked in the movie
	**Social Judgment & Salience**	*Negative Preferences*	Answer Typicality when asked about what they did not like in the movie
Vocal Prosody Measures		*Pitch*	Vocal Pitch (f0)
		Pitch Variation	Pitch variation (std(f0))
		Loudness	Vocal intensity in dB
		Loudness Variation	Intensity variation (std(dB))
		Dysphonia	Mean cepstral peak prominence
		Breathiness	Mean glottal-to-noise excitation ratio
Movement Measures		*Facial Movements*	Total movement in the video, where landmarks of interest were visible
		Eye Movements	Total movement in the video, where landmarks of interest were visible
		Mouth Movements	Total movement in the video, where landmarks of interest were visible
		Body Movements	Total movement in the video, where landmarks of interest were visible

Extended Data Figure S1. Validity of interview questions.

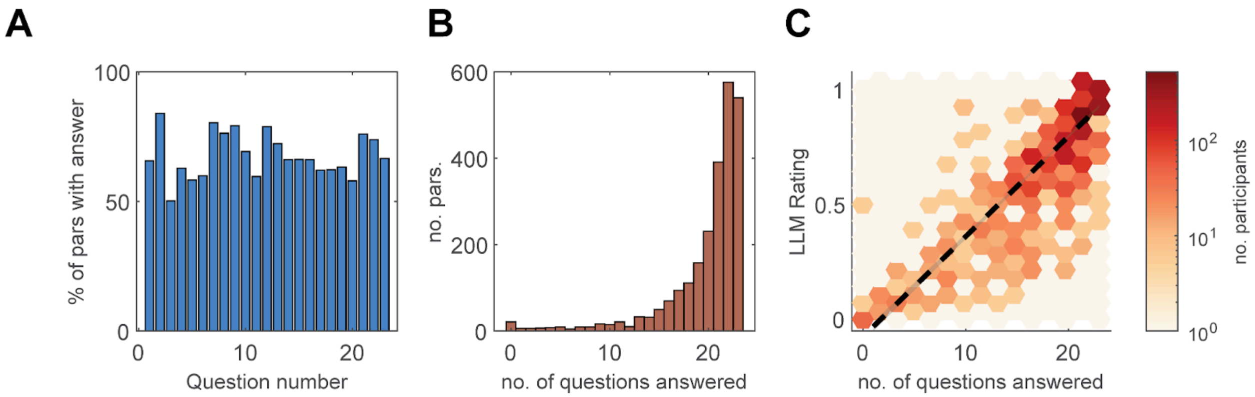

Extended Data Figure S1. Validity of interview questions. A) Not all questions were asked to every participant, or the answer was not available. In average, 67.7% of participants answered each question. B) Each participant answered 19.9 questions in average. C) The number of questions answered by each participant is correlated with the LLM's rating of the conversation completion ($\rho(2339) = 0.79$, $p<0.001$), see Supplementary Materials Section S3 for rating prompt.

bioRxiv preprint doi: https://doi.org/10.1101/2025.10.20.682864; this version posted October 20, 2025. The copyright holder for this preprint (which was not certified by peer review) is the author/funder, who has granted bioRxiv a license to display the preprint in perpetuity. It is made available under aCC-BY 4.0 International license.

Extended Data Figure S2. Distribution of Autism Scores in the ASD-positive population

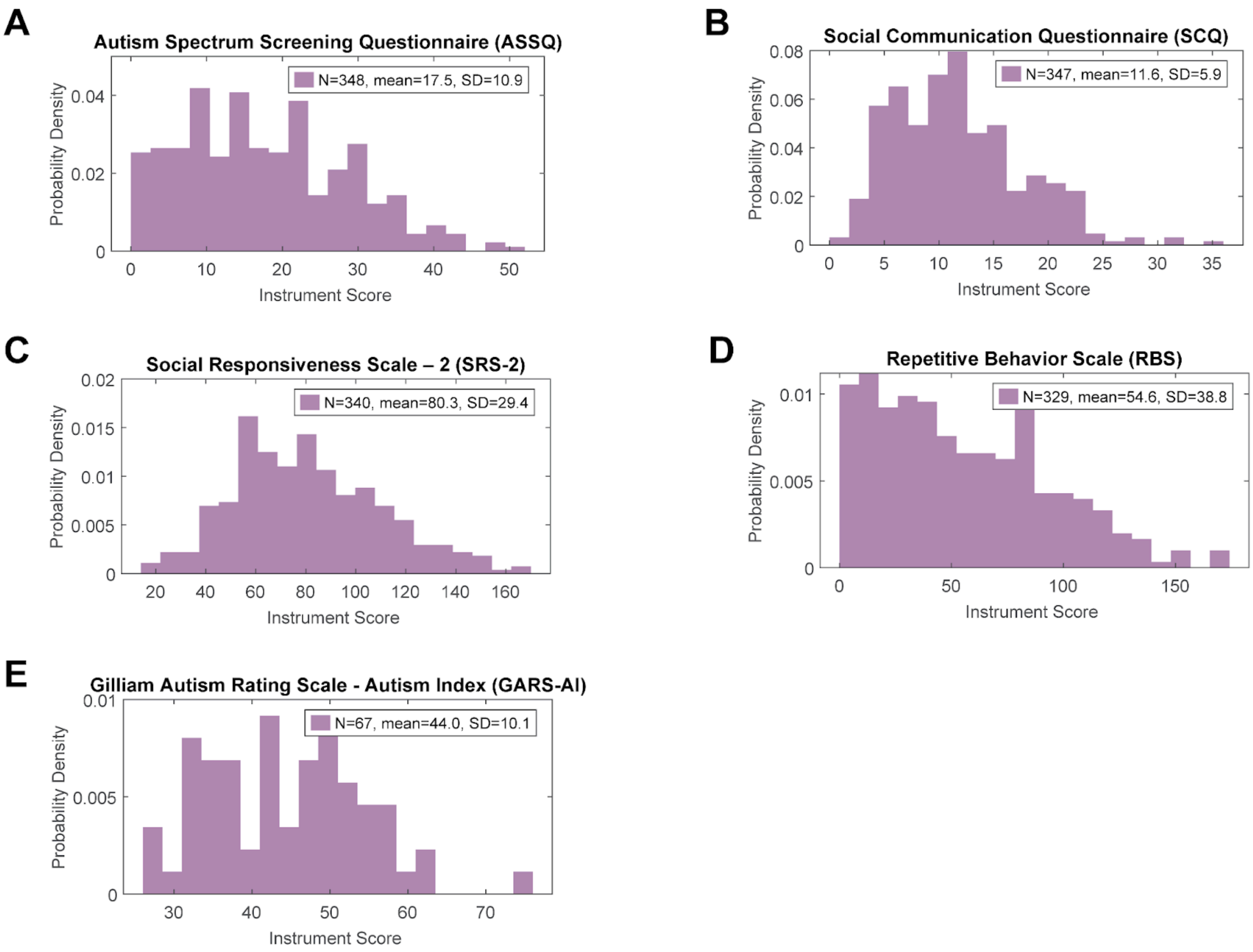

Extended Data Figure S2. Distribution of Autism Scores in the ASD-positive population. The Healthy Brain Network's deep phenotyping protocol provides multiple parent- and self-report questionnaires to measure traits associated with ASD. These included the Autism Spectrum Screening Questionnaire (ASSQ), the Social Communication Questionnaire (SCQ), the Social Responsiveness Scale-2 (SRS-2). The ASD population is comprised primarily of mild-to-moderate cases.

bioRxiv preprint doi: https://doi.org/10.1101/2025.10.20.682864; this version posted October 20, 2025. The copyright holder for this preprint (which was not certified by peer review) is the author/funder, who has granted bioRxiv a license to display the preprint in perpetuity. It is made available under aCC-BY 4.0 International license.

Extended Data Figure S3. Effect size matrices for primary behavioral outcomes after excluding IQ as covariate.

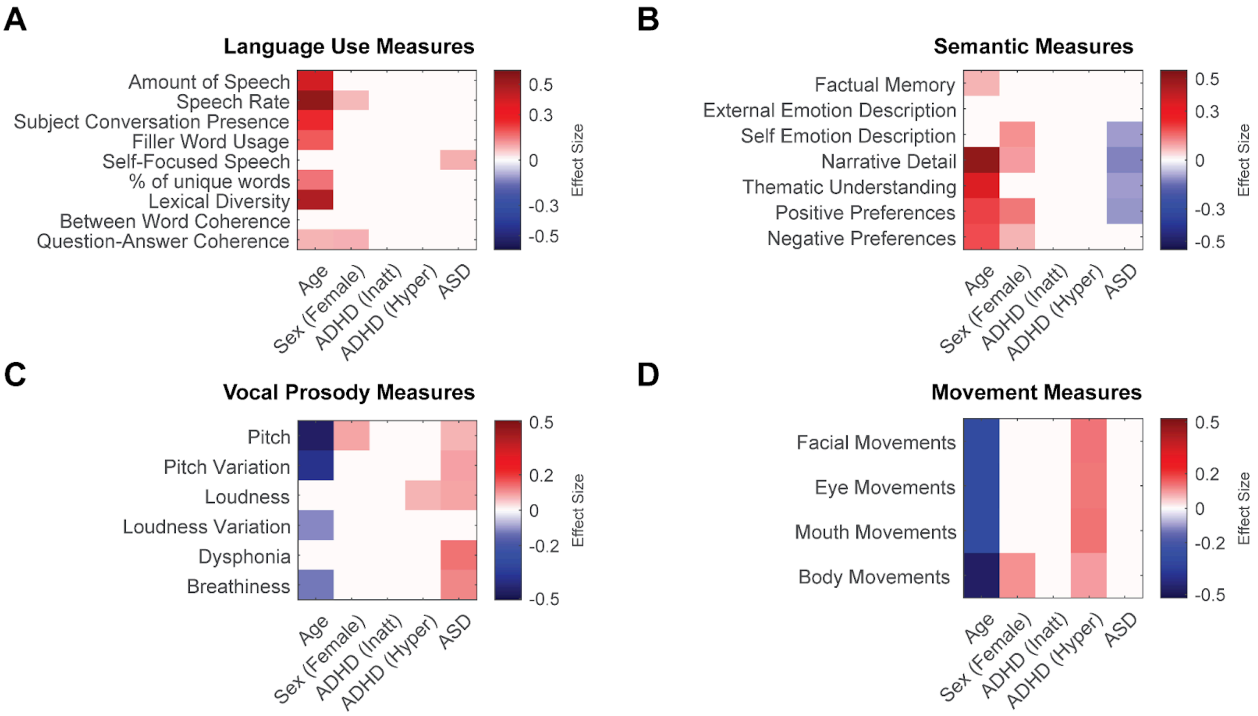

Extended Data Figure S3. Effect size matrices for primary behavioral outcomes after excluding IQ as covariate. The models (A through D) are identical to the primary multivariate models presented in the main text, but without modelling IQ as covariate. Results are mainly consistent. Effect sizes are estimated using multivariate regression, with p<0.01 after Bonferroni correction.

Extended Data Figure S4. Comparing Clinical Instruments to Measured Behavior.

bioRxiv preprint doi: https://doi.org/10.1101/2025.10.20.682864; this version posted October 20, 2025. The copyright holder for this preprint (which was not certified by peer review) is the author/funder, who has granted bioRxiv a license to display the preprint in perpetuity. It is made available under aCC-BY 4.0 International license.

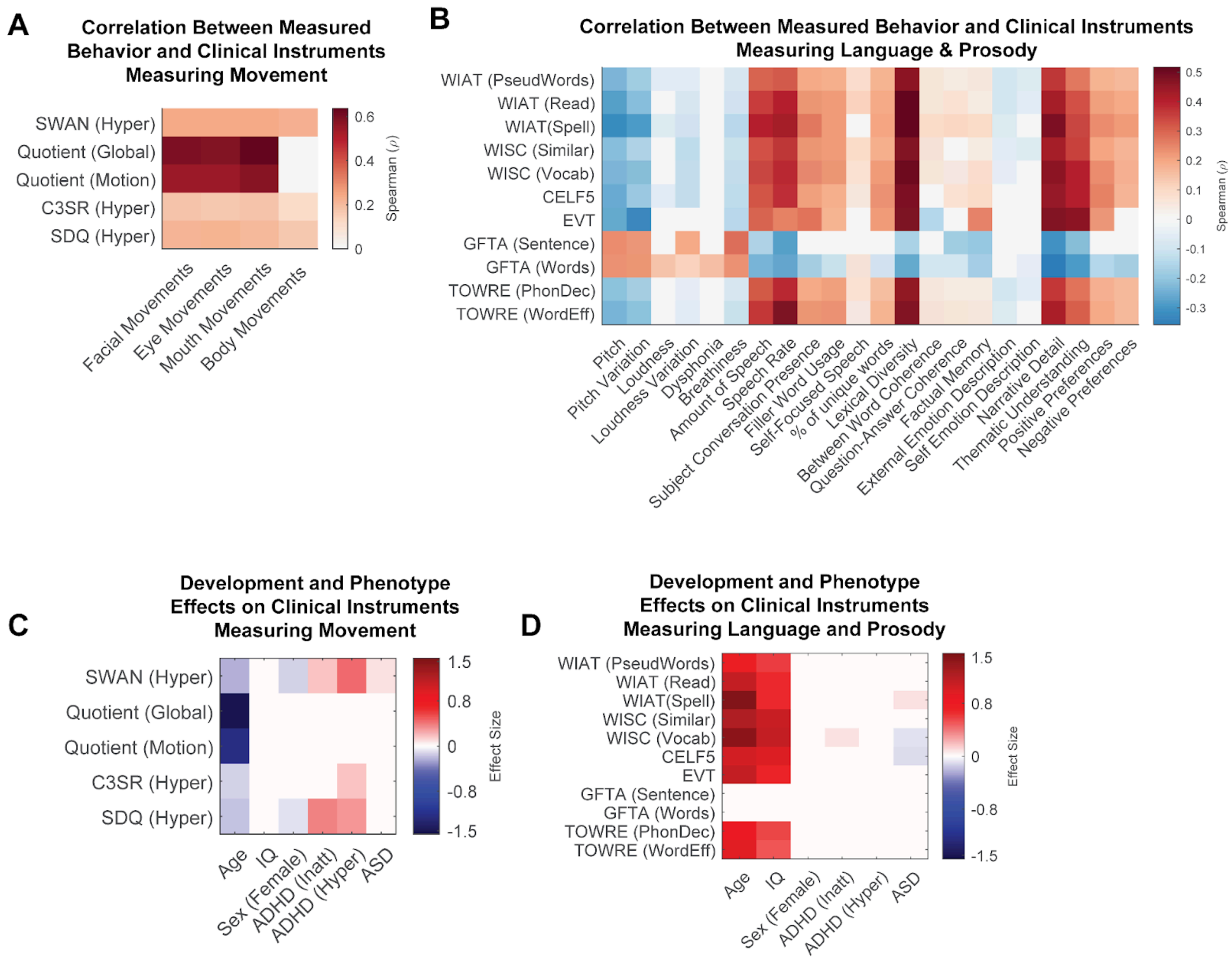

Extended Data Figure S4. Comparing Clinical Instruments to Measured Behavior. Spearman correlation between automatically measured behaviors and the clinical instrument scores measuring movement (A), and language use/prosody (B). C-D) Effects of development and diagnosis on such clinical instrument scores. As one may expect, our measures of movement, language and speech, correlate strongly with these established metrics (A, B). When using standard instruments it appears that ADHD-Inattention is also associated with elevated motor activity (C). Thus, our movement measures appear to be more specific to the ADHD-Hyperactive status (cf. main text Fig. 6C with panel C above). On the other hand, when using standard instruments, it appears that ASD has a lesser effect on language use (D). Therefore our features related to social communication appear to be more sensitive to the ASD presentation (cf. main text Fig. 4E with panel D above). Effect sizes in C, D measured using multivariate models, significance at $p<0.01$ after Bonferroni correction, white otherwise. For A and B, significant at a cutoff of $p<0.05$, uncorrected, white otherwise.

Extended Data Figure S5. Unique association of clinical questionnaire scales with clinician-confirmed diagnoses.

bioRxiv preprint doi: https://doi.org/10.1101/2025.10.20.682864; this version posted October 20, 2025. The copyright holder for this preprint (which was not certified by peer review) is the author/funder, who has granted bioRxiv a license to display the preprint in perpetuity. It is made available under aCC-BY 4.0 International license.

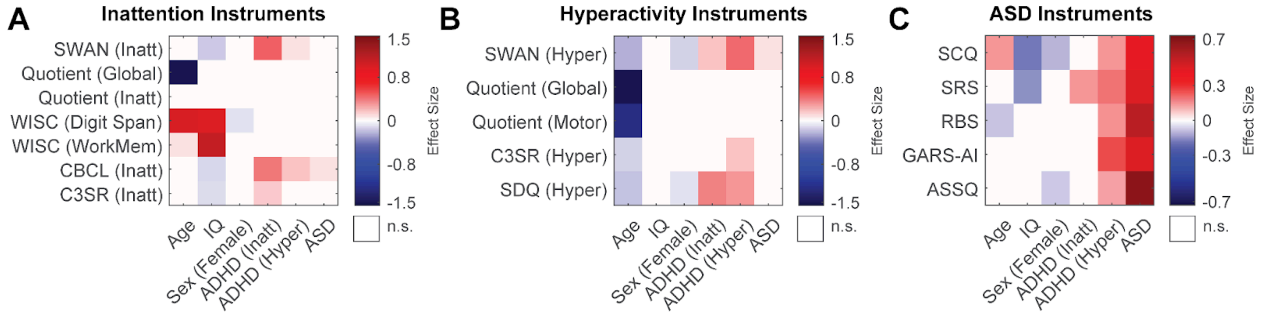

Extended Data Figure S5. Unique association of clinical questionnaire scales with clinician-confirmed diagnoses. The Healthy Brain Network's deep phenotyping protocol provides multiple parent- and self-report questionnaires to measure traits associated with ADHD and ASD.The effect size matrices reveal which clinical instruments are more uniquely explained by specific positive diagnoses while accounting for development and demographic effects. To select the most specific and robust trait measures for our analyses in main text Figures 2-3, we evaluated which of these scales best and most uniquely reflected the corresponding gold-standard, clinician-confirmed diagnosis. The SWAN Inattention and Hyperactivity subscales and the ASSQ emerged as the strongest and most specific associations for their corresponding clinician-confirmed diagnoses. Therefore, we selected these scales for all subsequent analyses where continuous measures of ADHD and ASD traits were required. Effect sizes measured using multivariate models, significance at p<0.01 after Bonferroni correction, white otherwise.

Extended Data Figure S6. Effect size matrices for primary behavioral outcomes after including anxiety as covariate.

(Figure S4).

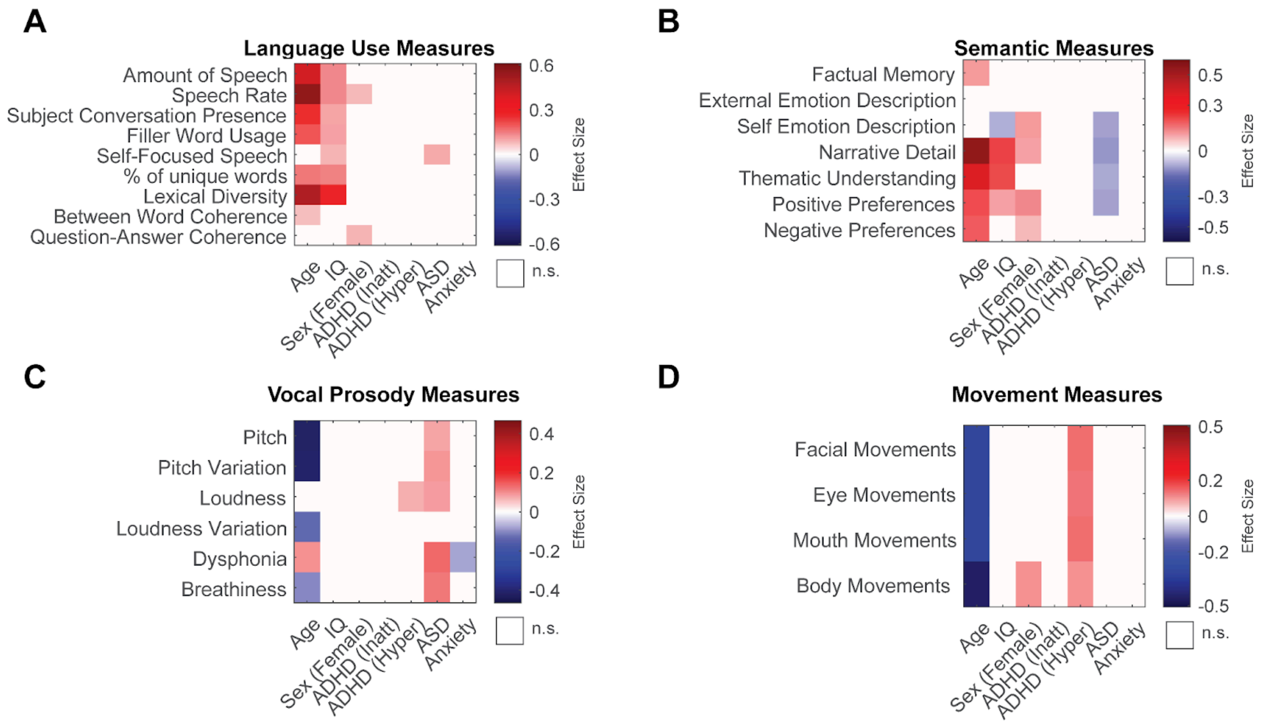

bioRxiv preprint doi: https://doi.org/10.1101/2025.10.20.682864; this version posted October 20, 2025. The copyright holder for this preprint (which was not certified by peer review) is the author/funder, who has granted bioRxiv a license to display the preprint in perpetuity. It is made available under aCC-BY 4.0 International license.

Extended Data Figure S6. Effect size matrices for primary behavioral outcomes after including anxiety as covariate. Given the high rates of comorbid anxiety in both ADHD and ASD, and the potential for a semi-structured interview with an unfamiliar clinician to elicit anxious behaviors, we modeled anxiety as a key potential confound. Behaviors associated with anxiety such as motor restlessness, social reticence, or altered vocal tone could overlap with the objective behavioral markers under investigation and potentially obscure the specific effects of ADHD and ASD. To address this and confirm the robustness of our primary findings, we repeated our primary multivariate regressions, adding a binary variable for any clinician-confirmed DSM-5 anxiety diagnosis as an additional covariate (N = 789). This allowed us to test whether the observed associations between our objective behavioral markers and ADHD/ASD diagnoses remained significant after statistically controlling for the influence of anxiety. We find that all the associations between ASD and/or ADHD with our behavioral measures are independent of whether we model anxiety or not. Effect sizes measured using multivariate models, significance at p<0.01 after Bonferroni correction, white otherwise.

Extended Data Figure S7. Results are independent of how the ADHD presentations are coded.

.

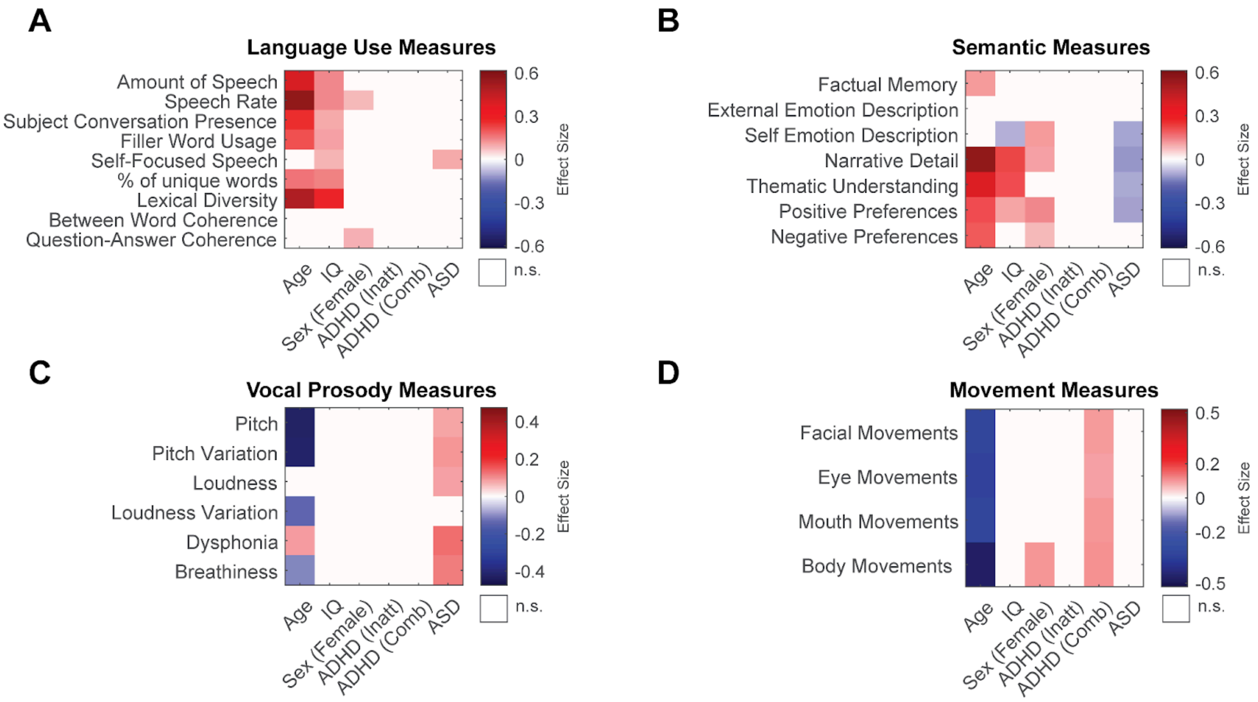

Extended Data Figure S7. Results are independent of how the ADHD presentations are coded. Throughout the work presented in this manuscript, clinician-diagnosed ADHD-Combined presentations were modelled as positive in both ADHD-Hyperactive and ADHD-Inattentive status. This was done to maximize the number of participants in the modelled group, while maintaining the distinction of the two main inattentive and hyperactive diagnostic status. Previous studies on the HBN population have modelled this overlap by considering the ADHD-Combined separate from the ADHD-Inattentive presentations [89–91]. We repeated the analyses on the behavioral features by following this approach, and the results were mostly consistent with our associations with the ADHD-Hyperactive presentation. The models (A through D) are identical to the primary multivariate models presented in the main text, but

bioRxiv preprint doi: https://doi.org/10.1101/2025.10.20.682864; this version posted October 20, 2025. The copyright holder for this preprint (which was not certified by peer review) is the author/funder, who has granted bioRxiv a license to display the preprint in perpetuity. It is made available under aCC-BY 4.0 International license.

ADHD modelled separately as the ADHD-Combined (N=625) and ADHD-Inattentive (N=653) presentations. Effect sizes are estimated using multivariate regression, with $p<0.01$ after Bonferroni correction.

bioRxiv preprint doi: https://doi.org/10.1101/2025.10.20.682864; this version posted October 20, 2025. The copyright holder for this preprint (which was not certified by peer review) is the author/funder, who has granted bioRxiv a license to display the preprint in perpetuity. It is made available under aCC-BY 4.0 International license.

Supplementary Material

Contents

Supplementary Materials Section S1. Multivariate Model Reports....................................39
Supplementary Materials Section S1.1. Diagnoses..39
Supplementary Materials Section S1.2. Clinical Instruments...................................... 39
Supplementary Materials Section S1.3. Language Use Measures............................... 41
Supplementary Materials Section S1.4. Semantic Measures.......................................44
Supplementary Materials Section S1.5. Vocal Prosody Measures................................46
Supplementary Materials Section S1.6. Movement Measures..................................... 48
Supplementary Materials Section S2. Diarizing Prompt for LLM...50
Supplementary Materials Section S2. Question-Answer Extraction Prompt for LLM........... 51

Supplementary Materials Section S1. Multivariate Model Reports.

p-values reported here appear uncorrected for multiple comparisons. For Figures 3-6, Effect Size matrices show significant effect sizes with a cutoff of p<0.01, white otherwise. p-values for the displays and analyses are Bonferroni-corrected for multiple comparisons by multiplying the model p-values (reported below) by the number of tested behavioral features.

Supplementary Materials Section S1.1. Diagnoses

Detailed model reports for panel A in Figure 2 and black arrows in panel C in Figure 3.

--- Results for Outcome: ASD ---

Predictor	Odds Ratio (OR)	95% CI	p-value
Age	1.033	[0.997, 1.07]	0.0746
Sex (Female)	0.426	[0.315, 0.575]	2.57E-08
ADHD (Inatt)	2.251	[1.69, 3]	3.55E-08
ADHD (Hyper)	1.597	[1.22, 2.08]	0.000548

--- Results for Outcome: ADHD (Inatt) ---

Predictor	Odds Ratio (OR)	95% CI	p-value
Age	1.071	[1.04, 1.1]	1.03E-06
Sex (Female)	0.681	[0.56, 0.828]	0.000118
ADHD (Hyper)	10.008	[7.8, 12.8]	1.38E-73
ASD	2.256	[1.69, 3.01]	3.62E-08

bioRxiv preprint doi: https://doi.org/10.1101/2025.10.20.682864; this version posted October 20, 2025. The copyright holder for this preprint (which was not certified by peer review) is the author/funder, who has granted bioRxiv a license to display the preprint in perpetuity. It is made available under aCC-BY 4.0 International license.

--- Results for Outcome: ADHD (Hyper) ---

Predictor	Odds Ratio (OR)	95% CI	p-value
Age	0.826	[0.797, 0.856]	7.84E-26
Sex (Female)	0.58	[0.46, 0.731]	3.90E-06
ADHD (Inatt)	10.46	[8.13, 13.5]	1.44E-74
ASD	1.601	[1.22, 2.09]	0.000593

Supplementary Materials Section S1.2. Clinical Instruments

Detailed model reports for panels E & F in Figure 2.

--- Results for Feature: Externalizing (CBCL) ---

Model Fit: $R^2 = 0.146$, Adjusted $R^2 = 0.144$

Overall Model: $F(5, 2126) = 72.513$, $p = 2.96e-70$

Predictor	Coefficient (β)	Std. Error	t-stat	Effect Size	p-value
Age	-0.0753	0.0584	-1.29	-0.028	0.197
Sex (Female)	-0.0637	0.39	-0.163	-0.00354	0.87
ADHD (Inatt)	1.48	0.407	3.63	0.0787	0.000292
ADHD (Hyper)	6.06	0.438	13.8	0.3	9.10E-42
ASD	0.298	0.517	0.577	0.0125	0.564

--- Results for Feature: Internalizing (CBCL) ---

Model Fit: $R^2 = 0.087$, Adjusted $R^2 = 0.085$

Overall Model: $F(5, 2126) = 40.583$, $p = 5.62e-40$

Predictor	Coefficient (β)	Std. Error	t-stat	Effect Size	p-value
Age	0.451	0.0501	8.98	0.195	5.57E-19
Sex (Female)	1.25	0.335	3.74	0.0811	1.91E-04
ADHD (Inatt)	1.16	0.35	3.31	0.0718	0.000951
ADHD (Hyper)	0.991	0.376	2.63	0.0571	0.00851
ASD	2.55	0.444	5.75	0.125	1.05E-08

--- Results for Feature: Anxiety Traits (SCARED) ---

Model Fit: $R^2 = 0.040$, Adjusted $R^2 = 0.038$

Overall Model: $F(5, 2142) = 17.822$, $p = 2.4e-17$

Predictor	Coefficient (β)	Std. Error	t-stat	Effect Size	p-value

bioRxiv preprint doi: https://doi.org/10.1101/2025.10.20.682864; this version posted October 20, 2025. The copyright holder for this preprint (which was not certified by peer review) is the author/funder, who has granted bioRxiv a license to display the preprint in perpetuity. It is made available under aCC-BY 4.0 International license.

Age	0.19	0.0701	2.71	0.0586	0.00677
Sex (Female)	2.36	0.496	4.76	0.103	2.04E-06
ADHD (Inatt)	1.1	0.515	2.13	0.046	0.0333
ADHD (Hyper)	0.411	0.556	0.739	0.016	0.46
ASD	2.32	0.648	3.58	0.0773	3.54E-04

--- Results for Feature: Depression Traits (MFQ) ---

Model Fit: $R^2 = 0.078$, Adjusted $R^2 = 0.076$

Overall Model: $F(5, 2151) = 36.627$, $p = 4.07e-36$

Predictor	Coefficient (β)	Std. Error	t-stat	Effect Size	p-value
Age	0.23	0.0464	4.97	0.107	7.33E-07
Sex (Female)	1.11	0.328	3.38	0.0729	0.000729
ADHD (Inatt)	1.82	0.341	5.34	0.115	1.00E-07
ADHD (Hyper)	1.33	0.368	3.62	0.0781	0.000299
ASD	1.01	0.429	2.35	0.0507	0.0187

Supplementary Materials Section S1.3. Language Use Measures

Detailed model reports for panels D & E in Figure 3.

--- Results for Feature: Amount of Speech ---

Model Fit: $R^2 = 0.154$, Adjusted $R^2 = 0.151$

Overall Model: $F(6, 2104) = 63.662$, $p = 8.01e-73$

Predictor	Coefficient (β)	Std. Error	t-stat	Effect Size	p-value
Age	0.0881	0.00483	18.3	0.398	2.98E-69
IQ	0.00529	0.000845	6.25	0.136	4.87E-10
Sex (Female)	0.0646	0.0302	2.14	0.0466	0.0327
ADHD (Inatt)	0.0435	0.0316	1.38	0.03	0.169
ADHD (Hyper)	0.00959	0.0341	0.281	0.00613	0.779
ASD	0.00618	0.04	0.154	0.00337	0.877

--- Results for Feature: Speech Rate ---

Model Fit: $R^2 = 0.251$, Adjusted $R^2 = 0.249$

Overall Model: $F(6, 2104) = 117.372$, $p = 4.34e-128$

Predictor	Coefficient (β)	Std. Error	t-stat	Effect Size	p-value
Age	5.49	0.212	25.8	0.563	6.74E-128

bioRxiv preprint doi: https://doi.org/10.1101/2025.10.20.682864; this version posted October 20, 2025. The copyright holder for this preprint (which was not certified by peer review) is the author/funder, who has granted bioRxiv a license to display the preprint in perpetuity. It is made available under aCC-BY 4.0 International license.

IQ	0.231	0.0372	6.21	0.135	6.32E-10
Sex (Female)	4.77	1.33	3.58	0.0781	0.000349
ADHD (Inatt)	-1.45	1.39	-1.04	-0.0228	0.297
ADHD (Hyper)	4.02	1.5	2.68	0.0583	0.0075
ASD	-3.28	1.76	-1.86	-0.0406	0.063

--- Results for Feature: Subject Conversation Presence ---

Model Fit: $R^2 = 0.072$, Adjusted $R^2 = 0.069$

Overall Model: $F(6, 2104) = 27.199$, $p = 2.19e-31$

**Predictor**	**Coefficient (β)**	**Std. Error**	**t-stat**	**Effect Size**	**p-value**
Age	1.33	0.113	11.7	0.256	8.63E-31
IQ	0.0914	0.0199	4.6	0.1	4.51E-06
Sex (Female)	1.11	0.711	1.56	0.034	0.119
ADHD (Inatt)	1.55	0.743	2.08	0.0454	0.0376
ADHD (Hyper)	0.0615	0.801	0.0768	0.00167	0.939
ASD	0.225	0.941	0.24	0.00522	0.811

--- Results for Feature: Filler Word Usage ---

Model Fit: $R^2 = 0.062$, Adjusted $R^2 = 0.060$

Overall Model: $F(6, 2122) = 23.512$, $p = 4.96e-27$

**Predictor**	**Coefficient (β)**	**Std. Error**	**t-stat**	**Effect Size**	**p-value**
Age	0.272	0.0282	9.65	0.21	1.33E-21
IQ	0.0251	0.00496	5.06	0.11	4.60E-07
Sex (Female)	0.0974	0.177	0.552	0.012	0.581
ADHD (Inatt)	0.0397	0.185	0.215	0.00466	0.83
ADHD (Hyper)	0.111	0.199	0.559	0.0121	0.576
ASD	-0.388	0.233	-1.66	-0.0361	0.0968

--- Results for Feature: Self-Focused Speech ---

Model Fit: $R^2 = 0.031$, Adjusted $R^2 = 0.029$

Overall Model: $F(6, 2100) = 11.306$, $p = 1.83e-12$

**Predictor**	**Coefficient (β)**	**Std. Error**	**t-stat**	**Effect Size**	**p-value**
Age	-0.0402	0.0977	-0.411	-0.00897	0.681

bioRxiv preprint doi: https://doi.org/10.1101/2025.10.20.682864; this version posted October 20, 2025. The copyright holder for this preprint (which was not certified by peer review) is the author/funder, who has granted bioRxiv a license to display the preprint in perpetuity. It is made available under aCC-BY 4.0 International license.

IQ	0.0669	0.0171	3.9	0.0852	9.78E-05
Sex (Female)	-1.23	0.613	-2	-0.0437	0.0456
ADHD (Inatt)	0.711	0.641	1.11	0.0242	0.267
ADHD (Hyper)	-0.313	0.691	-0.454	-0.0099	0.65
ASD	3.7	0.81	4.57	0.0996	5.28E-06

--- Results for Feature: % of unique words ---

Model Fit: $R^2 = 0.065$, Adjusted $R^2 = 0.062$

Overall Model: $F(6, 2104) = 24.246$, $p = 6.8e-28$

Predictor	Coefficient (β)	Std. Error	t-stat	Effect Size	p-value
Age	0.188	0.0255	7.35	0.16	2.93E-13
IQ	0.0298	0.00447	6.65	0.145	3.62E-11
Sex (Female)	0.446	0.16	2.79	0.0607	0.00538
ADHD (Inatt)	-0.294	0.167	-1.76	-0.0383	0.0788
ADHD (Hyper)	-0.243	0.18	-1.35	-0.0294	0.177
ASD	0.0827	0.212	0.39	0.00851	0.696

--- Results for Feature: Lexical Diversity ---

Model Fit: $R^2 = 0.246$, Adjusted $R^2 = 0.244$

Overall Model: $F(6, 2104) = 114.224$, $p = 5.05e-125$

Predictor	Coefficient (β)	Std. Error	t-stat	Effect Size	p-value
Age	0.00446	0.000199	22.4	0.489	6.63E-100
IQ	0.000437	3.49E-05	12.5	0.273	9.25E-35
Sex (Female)	0.00295	0.00125	2.37	0.0516	0.0181
ADHD (Inatt)	0.00242	0.00131	1.86	0.0405	0.0635
ADHD (Hyper)	-0.00282	0.00141	-2	-0.0437	0.0453
ASD	0.00149	0.00165	0.899	0.0196	0.369

--- Results for Feature: Between Word Coherence ---

Model Fit: $R^2 = 0.013$, Adjusted $R^2 = 0.010$

Overall Model: $F(6, 2104) = 4.709$, $p = 9.03e-05$

Predictor	Coefficient (β)	Std. Error	t-stat	Effect Size	p-value
Age	0.000141	4.30E-05	3.28	0.0714	0.00107
IQ	1.99E-06	7.53E-06	0.264	0.00576	0.791

bioRxiv preprint doi: https://doi.org/10.1101/2025.10.20.682864; this version posted October 20, 2025. The copyright holder for this preprint (which was not certified by peer review) is the author/funder, who has granted bioRxiv a license to display the preprint in perpetuity. It is made available under aCC-BY 4.0 International license.

Sex (Female)	0.000214	0.000269	0.793	0.0173	0.428
ADHD (Inatt)	-0.000485	0.000282	-1.72	-0.0375	0.0858
ADHD (Hyper)	1.73E-05	0.000304	0.0568	0.00124	0.955
ASD	-0.00104	0.000357	-2.92	-0.0637	0.00353

--- Results for Feature: Question-Answer Coherence ---

Model Fit: $R^2 = 0.014$, Adjusted $R^2 = 0.011$

Overall Model: $F(6, 2087) = 5.029$, $p = 3.96e-05$

Predictor	Coefficient (β)	Std. Error	t-stat	Effect Size	p-value
Age	0.00151	0.000526	2.87	0.0628	0.00416
IQ	-9.45E-06	9.20E-05	-0.103	-0.00225	0.918
Sex (Female)	0.0134	0.00329	4.08	0.0892	4.73E-05
ADHD (Inatt)	-0.00316	0.00344	-0.92	-0.0201	0.358
ADHD (Hyper)	0.00374	0.00371	1.01	0.0221	0.314
ASD	-0.0047	0.00435	-1.08	-0.0236	0.281

Supplementary Materials Section S1.4. Semantic Measures

Detailed model reports for panel E in Figure 4.

--- Results for Feature: Factual Memory ---

Model Fit: $R^2 = 0.018$, Adjusted $R^2 = 0.015$

Overall Model: $F(6, 2018) = 6.284$, $p = 1.48e-06$

Predictor	Coefficient (β)	Std. Error	t-stat	Effect Size	p-value
Age	0.0108	0.0021	5.13	0.114	3.11E-07
IQ	0.000716	0.00037	1.94	0.0431	0.0528
Sex (Female)	-0.0164	0.0132	-1.24	-0.0276	0.214
ADHD (Inatt)	0.00958	0.0138	0.694	0.0155	0.488
ADHD (Hyper)	-0.00847	0.0149	-0.57	-0.0127	0.569
ASD	-0.0435	0.0173	-2.51	-0.0559	0.0122

--- Results for Feature: External Emotion Description---

Model Fit: $R^2 = 0.005$, Adjusted $R^2 = 0.002$

Overall Model: $F(6, 2096) = 1.729$, $p = 0.11$

Predictor	Coefficient (β)	Std. Error	t-stat	Effect Size	p-value
Age	-0.00207	0.000879	-2.35	-0.0513	1.89E-02

bioRxiv preprint doi: https://doi.org/10.1101/2025.10.20.682864; this version posted October 20, 2025. The copyright holder for this preprint (which was not certified by peer review) is the author/funder, who has granted bioRxiv a license to display the preprint in perpetuity. It is made available under aCC-BY 4.0 International license.

IQ	5.99E-05	0.000155	0.387	0.00845	6.99E-01
Sex (Female)	0.00226	0.00551	0.41	0.00895	0.682
ADHD (Inatt)	0.0126	0.00577	2.19	0.0478	0.0289
ADHD (Hyper)	-0.00409	0.00622	-0.657	-0.0144	0.511
ASD	0.00165	0.00732	0.226	0.00494	0.821

--- Results for Feature: Self Emotion Description ---

Model Fit: $R^2 = 0.035$, Adjusted $R^2 = 0.032$

Overall Model: $F(6, 2080) = 12.671$, $p = 4.34e-14$

Predictor	Coefficient (β)	Std. Error	t-stat	Effect Size	p-value
Age	0.00156	0.00139	1.13	0.0247	2.60E-01
IQ	-0.000989	0.000244	-4.05	-0.0888	5.36E-05
Sex (Female)	0.0475	0.00872	5.45	0.119	5.67E-08
ADHD (Inatt)	0.000366	0.00911	0.0401	0.00088	0.968
ADHD (Hyper)	0.0197	0.00981	2	0.0439	0.0452
ASD	-0.0547	0.0116	-4.74	-0.104	2.32E-06

--- Results for Feature: Narrative Detail ---

Model Fit: $R^2 = 0.282$, Adjusted $R^2 = 0.280$

Overall Model: $F(6, 2084) = 136.422$, $p = 5.2e-146$

Predictor	Coefficient (β)	Std. Error	t-stat	Effect Size	p-value
Age	0.0439	0.00174	25.2	0.552	1.53E-122
IQ	0.0031	0.000305	10.2	0.223	8.02E-24
Sex (Female)	0.0509	0.0108	4.69	0.103	2.89E-06
ADHD (Inatt)	0.0324	0.0113	2.86	0.0626	0.00428
ADHD (Hyper)	-0.0327	0.0122	-2.67	-0.0584	0.0077
ASD	-0.0765	0.0144	-5.32	-0.117	1.13E-07

--- Results for Feature: Thematic Understanding ---

Model Fit: $R^2 = 0.164$, Adjusted $R^2 = 0.161$

Overall Model: $F(6, 1859) = 60.793$, $p = 5.6e-69$

Predictor	Coefficient (β)	Std. Error	t-stat	Effect Size	p-value
Age	0.0647	0.00392	16.5	0.383	3.29E-57

bioRxiv preprint doi: https://doi.org/10.1101/2025.10.20.682864; this version posted October 20, 2025. The copyright holder for this preprint (which was not certified by peer review) is the author/funder, who has granted bioRxiv a license to display the preprint in perpetuity. It is made available under aCC-BY 4.0 International license.

IQ	0.00645	0.000699	9.23	0.214	7.18E-20
Sex (Female)	-0.0216	0.0244	-0.883	-0.0205	0.377
ADHD (Inatt)	0.0368	0.0257	1.43	0.0331	0.153
ADHD (Hyper)	-0.039	0.0278	-1.4	-0.0325	0.161
ASD	-0.136	0.0325	-4.2	-0.0973	2.84E-05

--- Results for Feature: Positive Preferences ---

Model Fit: R^2 = 0.087, Adjusted R^2 = 0.084

Overall Model: F(6, 1867) = 29.728, p = 3.47e-34

Predictor	Coefficient (β)	Std. Error	t-stat	Effect Size	p-value
Age	0.0144	0.00158	9.09	0.21	2.51E-19
IQ	1.25E-03	0.00028	4.47	0.103	8.30E-06
Sex (Female)	0.0596	0.01	5.94	0.137	3.40E-09
ADHD (Inatt)	0.00264	0.0106	0.25	0.00579	0.803
ADHD (Hyper)	-0.00982	0.0114	-0.863	-0.02	0.388
ASD	-0.061	0.0133	-4.6	-0.106	4.57E-06

--- Results for Feature: Negative Preferences---

Model Fit: R^2 = 0.050, Adjusted R^2 = 0.047

Overall Model: F(6, 1798) = 15.771, p = 9.98e-18

Predictor	Coefficient (β)	Std. Error	t-stat	Effect Size	p-value
Age	0.0108	0.00132	8.14	0.192	7.32E-16
IQ	0.000406	0.000234	1.73	0.0408	0.0836
Sex (Female)	0.0272	0.00839	3.24	0.0764	0.00121
ADHD (Inatt)	-0.00306	0.00879	-0.348	-0.00821	0.728
ADHD (Hyper)	-0.0106	0.00948	-1.11	-0.0263	0.266
ASD	-0.0158	0.011	-1.43	-0.0338	0.152

Supplementary Materials Section S1.5. Vocal Prosody Measures

Detailed model reports for panel B in Figure 5.

--- Results for Feature: Pitch ---

Model Fit: R^2 = 0.172, Adjusted R^2 = 0.170

Overall Model: F(6, 2109) = 72.945, p = 7.27e-83

bioRxiv preprint doi: https://doi.org/10.1101/2025.10.20.682864; this version posted October 20, 2025. The copyright holder for this preprint (which was not certified by peer review) is the author/funder, who has granted bioRxiv a license to display the preprint in perpetuity. It is made available under aCC-BY 4.0 International license.

Predictor	Coefficient (β)	Std. Error	t-stat	Effect Size	p-value
Age	-5.99	0.312	-19.2	-0.419	4.06E-76
IQ	-0.016	0.0547	-0.292	-0.00636	0.77
Sex (Female)	4.33	1.95	2.22	0.0483	0.0266
ADHD (Inatt)	0.198	2.04	0.0971	0.00211	0.923
ADHD (Hyper)	6.1	2.2	2.78	0.0605	0.00554
ASD	9.41	2.58	3.65	0.0794	0.000271

--- Results for Feature: Pitch Variation---

Model Fit: $R^2 = 0.177$, Adjusted $R^2 = 0.174$

Overall Model: $F(6, 2109) = 75.459$, $p = 1.52e-85$

Predictor	Coefficient (β)	Std. Error	t-stat	Effect Size	p-value
Age	-2.41	0.126	-19.1	-0.415	7.72E-75
IQ	0.0318	0.0222	1.44	0.0313	1.51E-01
Sex (Female)	1.47	0.791	1.86	0.0405	0.0633
ADHD (Inatt)	0.479	0.827	0.58	0.0126	0.562
ADHD (Hyper)	2.52	0.891	2.83	0.0616	4.72E-03
ASD	4.5	1.05	4.3	0.0937	1.77E-05

--- Results for Feature: Loudness---

Model Fit: $R^2 = 0.021$, Adjusted $R^2 = 0.018$

Overall Model: $F(6, 2109) = 7.474$, $p = 6.11e-08$

Predictor	Coefficient (β)	Std. Error	t-stat	Effect Size	p-value
Age	0.0745	0.0461	1.62	0.0352	0.106
IQ	-0.0169	0.00808	-2.09	-0.0456	0.0364
Sex (Female)	-0.684	0.288	-2.37	-0.0516	0.0178
ADHD (Inatt)	-0.767	0.302	-2.54	-0.0554	0.0111
ADHD (Hyper)	1.05	0.325	3.23	0.0702	1.28E-03
ASD	1.45	0.381	3.8	0.0828	1.49E-04

--- Results for Feature: Loudness Variation---

Model Fit: $R^2 = 0.029$, Adjusted $R^2 = 0.026$

Overall Model: $F(6, 2109) = 10.384$, $p = 2.27e-11$

bioRxiv preprint doi: https://doi.org/10.1101/2025.10.20.682864; this version posted October 20, 2025. The copyright holder for this preprint (which was not certified by peer review) is the author/funder, who has granted bioRxiv a license to display the preprint in perpetuity. It is made available under aCC-BY 4.0 International license.

Predictor	Coefficient (β)	Std. Error	t-stat	Effect Size	p-value
Age	-0.0922	0.0143	-6.44	-0.14	1.45E-10
IQ	-0.00428	0.00251	-1.7	-0.0371	0.0887
Sex (Female)	-0.274	0.0896	-3.06	-0.0666	0.00226
ADHD (Inatt)	0.0185	0.0937	0.197	0.00429	0.844
ADHD (Hyper)	-0.00554	0.101	-0.0549	-0.00119	0.956
ASD	0.26	0.118	2.2	0.0479	0.0281

--- Results for Feature: Dysphonia---

Model Fit: $R^2 = 0.052$, Adjusted $R^2 = 0.050$

Overall Model: $F(6, 2109) = 19.360$, $p = 4.39e-22$

Predictor	Coefficient (β)	Std. Error	t-stat	Effect Size	p-value
Age	0.0134	0.00341	3.94	0.0857	8.54E-05
IQ	-0.00104	0.000598	-1.74	-0.0378	0.0824
Sex (Female)	-0.0243	0.0213	-1.14	-0.0248	0.255
ADHD (Inatt)	-0.0284	0.0223	-1.27	-0.0278	0.203
ADHD (Hyper)	0.0175	0.024	0.729	0.0159	4.66E-01
ASD	0.172	0.0282	6.1	0.133	1.26E-09

--- Results for Feature: Breathiness---

Model Fit: $R^2 = 0.028$, Adjusted $R^2 = 0.025$

Overall Model: $F(6, 2109) = 10.032$, $p = 5.94e-11$

Predictor	Coefficient (β)	Std. Error	t-stat	Effect Size	p-value
Age	-0.00235	0.000495	-4.76	-0.104	2.11E-06
IQ	-6.02E-05	8.68E-05	-0.694	-0.0151	0.488
Sex (Female)	0.00597	0.0031	1.93	0.042	0.0541
ADHD (Inatt)	-0.00432	0.00324	-1.33	-0.029	0.183
ADHD (Hyper)	0.00247	0.00349	0.707	0.0154	4.80E-01
ASD	0.0219	0.00409	5.36	0.117	9.46E-08

Supplementary Materials Section S1.6. Movement Measures

Detailed model reports for panel B in Figure 6.

--- Results for Feature: overall_mean ---

bioRxiv preprint doi: https://doi.org/10.1101/2025.10.20.682864; this version posted October 20, 2025. The copyright holder for this preprint (which was not certified by peer review) is the author/funder, who has granted bioRxiv a license to display the preprint in perpetuity. It is made available under aCC-BY 4.0 International license.

Model Fit: $R^2 = 0.136$, Adjusted $R^2 = 0.134$

Overall Model: $F(6, 2141) = 56.376$, $p = 6.9e-65$

Predictor	Coefficient (β)	Std. Error	t-stat	Effect Size	p-value
Age	-0.0474	0.00323	-14.7	-0.317	1.93E-46
IQ	-0.000256	0.000568	-0.452	-0.00976	0.652
Sex (Female)	-0.026	0.0202	-1.29	-0.0278	0.199
ADHD (Inatt)	-0.0177	0.0212	-0.835	-0.018	4.04E-01
ADHD (Hyper)	0.147	0.0229	6.41	0.139	1.80E-10
ASD	0.0739	0.0267	2.76	0.0598	0.00575

--- Results for Feature: eyes_mean ---

Model Fit: $R^2 = 0.142$, Adjusted $R^2 = 0.140$

Overall Model: $F(6, 2141) = 59.035$, $p = 7.78e-68$

Predictor	Coefficient (β)	Std. Error	t-stat	Effect Size	p-value
Age	-0.0489	0.00328	-14.9	-0.322	7.28E-48
IQ	-0.000178	0.000576	-0.309	-0.00669	7.57E-01
Sex (Female)	-0.0551	0.0205	-2.69	-0.058	0.0073
ADHD (Inatt)	-0.0224	0.0215	-1.04	-0.0225	2.97E-01
ADHD (Hyper)	0.143	0.0232	6.14	0.133	1.01E-09
ASD	0.0794	0.0271	2.93	0.0633	3.46E-03

--- Results for Feature: lips_mean ---

Model Fit: $R^2 = 0.130$, Adjusted $R^2 = 0.127$

Overall Model: $F(6, 2141) = 53.259$, $p = 2.07e-61$

bioRxiv preprint doi: https://doi.org/10.1101/2025.10.20.682864; this version posted October 20, 2025. The copyright holder for this preprint (which was not certified by peer review) is the author/funder, who has granted bioRxiv a license to display the preprint in perpetuity. It is made available under aCC-BY 4.0 International license.

Predictor	Coefficient (β)	Std. Error	t-stat	Effect Size	p-value
Age	-0.0449	0.00315	-14.3	-0.308	3.53E-44
IQ	-0.000272	0.000553	-0.492	-0.0106	0.623
Sex (Female)	-0.00655	0.0197	-0.333	-0.00719	7.39E-01
ADHD (Inatt)	-0.0135	0.0206	-0.654	-0.0141	5.13E-01
ADHD (Hyper)	0.145	0.0223	6.49	0.14	1.03E-10
ASD	0.0686	0.026	2.64	0.057	0.00845

--- Results for Feature: overall_mean_pose ---

Model Fit: $R^2 = 0.209$, Adjusted $R^2 = 0.207$

Overall Model: $F(6, 2155) = 95.086$, $p = 3.18e-106$

Predictor	Coefficient (β)	Std. Error	t-stat	Effect Size	p-value
Age	-0.06	0.0028	-21.4	-0.461	2.65E-92
IQ	-0.0013	0.000492	-2.65	-0.057	8.18E-03
Sex (Female)	0.0855	0.0175	4.88	0.105	1.14E-06
ADHD (Inatt)	0.0139	0.0184	0.756	0.0163	4.50E-01
ADHD (Hyper)	0.0967	0.0198	4.88	0.105	1.13E-06

Supplementary Materials Section S2. Diarizing Prompt for LLM

```
""You are given a transcript of an interview between a clinician (interviewer) and a subject. The interviewer asks
questions from a predefined list, though slight variations may occur. Your task is to label the dialogue as either
"Interviewer:" or "Subject:", removing timestamps while preserving the dialogue structure.

### Instructions:
1. Identify lines spoken by the **interviewer** based on a predefined question list. These questions may have slight
variations but follow the same general intent.
2. Label all other responses as **"Subject:"**
3. Remove timestamps but keep the text structure intact.
4. Ensure proper formatting with a new line between each labeled turn.

### Example Input:

0.709 -> 2.83: I hope you enjoyed the last movie about the puppy.
2.95 -> 3.851: Have you seen it before?
4.451 -> 4.751: No.
5.231 -> 6.792: Can you tell me what happened in the movie?
6.852 -> 8.013: Try to tell the whole story.
8.093 -> 11.515: Remember that stories have a beginning, things that happen, and an ending.
12.895 -> 15.877: I didn't watch the whole thing though.
```

bioRxiv preprint doi: https://doi.org/10.1101/2025.10.20.682864; this version posted October 20, 2025. The copyright holder for this preprint (which was not certified by peer review) is the author/funder, who has granted bioRxiv a license to display the preprint in perpetuity. It is made available under aCC-BY 4.0 International license.

```
16.637 -> 18.478: Can you tell me about the part that you did watch?
19.139 -> 19.639: Okay.

### Example Output:

Interviewer: I hope you enjoyed the last movie about the puppy.
Interviewer: Have you seen it before?
Subject: No.
Interviewer: Can you tell me what happened in the movie?
Interviewer: Try to tell the whole story.
Interviewer: Remember that stories have a beginning, things that happen, and an ending.
Subject: I didn't watch the whole thing though.
Interviewer: Can you tell me about the part that you did watch?
Subject: Okay.

### If the transcript contains these specific sentences, you will label them as "clip" instead of interviewer or subject:

Whoa! Cool!
You gotta be kidding me
Get lost
Mom, I'll/we'll be outside

### The interviewer question list:

So I hope you enjoyed the last movie. Have you seen it before?
So can you tell me what happened in the movie? Try to tell the whole story. Remember that stories have a beginning,
things that happen, and an ending.
Do you remember anything else from the story?
So what are some of the things you liked about the movie?
What are some of the things you didn't like about the movie?
Who gave the boy a box?
What was in the box?
What was the boy doing before he got the box?
What was the puppy playing with?
How are the puppy and the boy the same?
So in the movie, who is missing a leg? The boy, the puppy, both the boy and the puppy, or no one?
So we're going to watch a short clip from the movie and then we'll talk about it.
How do you think the puppy was feeling?
How do you think the boy was feeling?
And how did you feel while you were watching that part?
How do you think the puppy was feeling?
How do you think the boy was feeling?
And how did you feel while you were watching that part?
How do you think the puppy was feeling?
How do you think the boy was feeling?
And how did you feel while you were watching that part?
How do you think the puppy was feeling?
How do you think the boy was feeling?
And how did you feel while you were watching this part?
Great, thank you

### In some cases, the interviewer may ask additional questions or make comments. You should label these as
"Interviewer:" as well using their specific context.

### The transcript:
<<transcript>>
```

bioRxiv preprint doi: https://doi.org/10.1101/2025.10.20.682864; this version posted October 20, 2025. The copyright holder for this preprint (which was not certified by peer review) is the author/funder, who has granted bioRxiv a license to display the preprint in perpetuity. It is made available under aCC-BY 4.0 International license.

Supplementary Materials Section S2. Question-Answer Extraction Prompt for LLM

```
"""Task: Extract Answers from Interview Transcript

You are tasked with extracting the participant's answers to a specific set of predefined questions from an interview
transcript. The questions relate to the short film "The Present." The interviewer *may* have asked these questions
verbatim, asked them in a slightly different way, or not asked them at all. The subject may have answered directly,
indirectly, partially, over multiple turns, or not at all.
In the transcript the interviewer asks these eleven questions in order:
1.  "I hope you enjoyed the last movie. Have you seen it before?"
2.  "Can you tell me what happened in the movie? Try to tell the whole story. Remember that stories have a beginning,
things that happen, and an ending."
3.  "Do you remember anything else from the story?"
4.  "What are some of the things you liked about the movie?"
5.  "What are some of the things you didn't like about the movie?"
6.  "Who gave the boy a box?"
7.  "What was in the box?"
8.  "What was the boy doing before he got the box?"
9.  "What was the puppy playing with?"
10. "How are the puppy and the boy the same?"
11. "In the movie, who is missing a leg? The boy, the puppy, both the boy and the puppy, or no one?"

After these questions, the interviewer then shows the subject four short clips from the movie and asks the subject to
describe how the characters are feeling in each clip. The interviewer may say something like:
- "Let's watch a short clip from the movie, and then we'll talk about it."
- "Now let's watch another clip"
- "One more clip"
- "One last clip"

After watching each clip, the interviewer asks the subject how they think the characters are feeling and how they
themselves feel while watching the clip. The interviewer may ask these questions in various ways, such as:
- "How do you think the puppy was feeling?"
- "How do you think the boy was feeling?"
- "And how did you feel while you were watching that part?"


Input:

*   A transcript of a conversation between an interviewer and a subject discussing the short film "The Present."

Output:

Your output will consist of two parts:

**Part 1: Question-Answer Extraction**

A series of lines, one for each predefined question. Each line will follow this format:

`"Question" -> "Answer"`

Where:

*   `"Question"` is one of the predefined questions listed below (use the *exact* wording provided here, even if the
interviewer phrased it differently).
*   `"Answer"` is the subject's answer to the question.
```

bioRxiv preprint doi: https://doi.org/10.1101/2025.10.20.682864; this version posted October 20, 2025. The copyright holder for this preprint (which was not certified by peer review) is the author/funder, who has granted bioRxiv a license to display the preprint in perpetuity. It is made available under aCC-BY 4.0 International license.

```
    *   If the question was answered (directly or indirectly), provide the subject's complete answer, concatenating all relevant parts of their response, even if it spans multiple turns or requires piecing together information from different parts of the transcript.
    *   If the question was *not* asked, or if the subject provided *no* discernible answer (even after considering the surrounding context), leave the "Answer" field *blank*. Do *not* write "N/A," "No answer," or any other placeholder. Just leave it blank.
    * If parts of different answers are mixed, extract only the answer to the intended question.

Predefined Questions:

1.  "I hope you enjoyed the last movie. Have you seen it before?"
2.  "Can you tell me what happened in the movie? Try to tell the whole story. Remember that stories have a beginning, things that happen, and an ending."
3.  "Do you remember anything else from the story?"
4.  "What are some of the things you liked about the movie?"
5.  "What are some of the things you didn't like about the movie?"
6.  "Who gave the boy a box?"
7.  "What was in the box?"
8.  "What was the boy doing before he got the box?"
9.  "What was the puppy playing with?"
10. "How are the puppy and the boy the same?"
11. "In the movie, who is missing a leg? The boy, the puppy, both the boy and the puppy, or no one?"
12. "How do you think the puppy was feeling? (after watching first clip)"
13. "How do you think the boy was feeling? (after watching first clip)"
14. "And how did you feel while you were watching that part? (after watching first clip)"
15. "How do you think the puppy was feeling? (after watching second clip)"
16. "How do you think the boy was feeling? (after watching second clip)"
17. "And how did you feel while you were watching that part? (after watching second clip)"
18. "How do you think the puppy was feeling? (after watching third clip)"
19. "How do you think the boy was feeling? (after watching third clip)"
20. "And how did you feel while you were watching that part? (after watching third clip)"
21. "How do you think the puppy was feeling? (after watching fourth clip)"
22. "How do you think the boy was feeling? (after watching fourth clip)"
23. "And how did you feel while you were watching that part? (after watching fourth clip)"

**Part 2: Conversation Completion Rating**

A single line in the following format:

`"Conversation Completion Rating" -> "Rating"`

Where:

*   `"Rating"` is a numerical score between 0 and 1 (inclusive) representing the overall completion of the conversation, *specifically with respect to the predefined questions*.
    *   **1:** Represents a perfect completion, where all of the predefined questions were asked (or closely paraphrased), and the subject provided clear and complete answers to each.
    *   **0:** Represents a very poor completion, where many questions were not asked, or the subject consistently failed to answer the questions that were asked.
    *   **Intermediate values:** Represent varying degrees of completion quality.  A score of 0.5, for example, would suggest that roughly half of the questions were asked and answered reasonably well. Use your judgment to assign a score that reflects the overall completeness and clarity of the question-answer exchange related to the *predefined questions*. Do *not* assess the overall quality of the conversation beyond its adherence to addressing the core content of these questions.

Instructions:
```

bioRxiv preprint doi: https://doi.org/10.1101/2025.10.20.682864; this version posted October 20, 2025. The copyright holder for this preprint (which was not certified by peer review) is the author/funder, who has granted bioRxiv a license to display the preprint in perpetuity. It is made available under aCC-BY 4.0 International license.

```
1.  **Read the Entire Transcript:** Carefully read the entire transcript to understand the completion of the conversation
and the context of each statement.
2.  **Identify Questions and Answers:** For each predefined question:
    *   **Search for the Question:** Look for the exact question, or a close paraphrase, in the interviewer's speech.
Note that the interviewer might rephrase, prompt, or interrupt the subject.
    *   **Locate the Answer (If Present):** If the question (or a close variant) was asked, carefully examine the
subject's subsequent responses. The answer might be:
        *   **Direct and Immediate:** Right after the question.
        *   **Indirect:** Implied by their statements, requiring inference.
        *   **Partial:** Only part of the answer is given directly.
        *   **Scattered:** Pieces of the answer are given across multiple turns, possibly with interviewer prompts or
interruptions.
        *   **Non-existent:** The subject might not answer at all.
    * **Use Context:** Consider surrounding conversational turns from both interviewer and subject to determine if a
particular utterance constitutes an answer, even an indirect or partial one.
    * **Combine Response Parts:** If the answer is spread over multiple turns, concatenate *all relevant parts* of the
subject's response into a single, coherent answer. Remove any interviewer interjections or prompts from within the
concatenated answer. Only include the *subject's* words in the "Answer" field.
4.  * **For the questions after the clips:** Because there are multiple clips, you will need to extract the answers to
these questions from the conversation after each clip is shown. The interviewer will ask the subject how they think the
characters are feeling and how they themselves feel while watching the clip. However, the order of the clips is always
the same. Sometimes part of the dialogue is present on the transcript, so in those cases, use that as clue to know to
what clip the question is referring to. If you can't clearly now what question/answer corresponds to what clip, restrain
from returning and answer for that specific question, leave the "Answer" field blank.:
    * **Clip 1:**: the kid opens the present and sees the puppy, the transcript might say "Whoa, cool."
    * **Clip 2:**: the kid throws the puppy away, the transcript might say "You've got to be kidding me."
    * **Clip 3:**: the kid kicks the puppy, the transcript might say "Get lost!"
    * **Clip 4:**: the kid plays with the puppy, the transcript might say "Mom, I'll / We'll be outside."
3.  **Output (Part 1):** Generate the question-answer extraction in the specified `"Question" -> "Answer"` format. Leave
the "Answer" field blank if the question was not asked or if no answer can be found.
4. **Assess Conversation Completion (Part 2):** After extracting the question-answer pairs, evaluate the *overall
completion* of the conversation *with respect to the predefined questions*. Consider:
    *  How many of the predefined questions (or close paraphrases) were asked by the interviewer?
    *  Did the conversation stay focused on the topics covered by the predefined questions, or did it frequently deviate?
5.  **Output (Part 2):** Provide the "Conversation Completion Rating" on a separate line, using a number between 0 and 1.

Example 1:

    (example 1 transcript):
        Interviewer: So I hope you enjoyed the last movie.
        Interviewer: Have you seen it before?
        Subject: I have at the mug MRI.
        Interviewer: Oh, cool.
        Subject: I liked it, though.
        Interviewer: Great.
        Interviewer: So can you tell me what happened in the movie?
        Interviewer: Try to tell the whole story.
        Interviewer: Remember that stories have a beginning, things that happen, and an ending.
        Subject: So there's a boy who was disabled, and he didn't want to go outside.
        Subject: He wasn't in the mood for anything except for his video games.
        Subject: and then like his mom was trying to like help him go like get some fresh air and then but like
everything like his mom tried to do didn't work so then he then she bought um him a puppy so then he started like he
didn't want like to see the puppy like anymore because then he realized he realized that he was also like disabled
        Subject: But then he started having a liking to him because he felt like the puppy and him were like the same.
        Subject: So he started playing with the puppy more.
        Subject: So then they went outside and they started playing with the ball.
        Interviewer: Great.
        Interviewer: Do you remember anything else from the story?
```

bioRxiv preprint doi: https://doi.org/10.1101/2025.10.20.682864; this version posted October 20, 2025. The copyright holder for this preprint (which was not certified by peer review) is the author/funder, who has granted bioRxiv a license to display the preprint in perpetuity. It is made available under aCC-BY 4.0 International license.

```
        Subject: I think that's much it.
        Interviewer: Great.
        Interviewer: So what are some of the things you liked about the movie?
        Subject: I liked how he had a good relationship with his doll.
        Interviewer: What are some of the things you didn't like about the movie?
        Subject: I liked everything about the movie, but
        Subject: I was hoping it would be longer.
        Interviewer: Who gave the boy a box?
        Subject: His mother.
        Interviewer: What was in the box?
        Subject: The puppy.
        Interviewer: What was the boy doing before he got the box?
        Subject: He wasn't really paying attention to anything.
        Subject: He was glued to his video games.
        Interviewer: What was the puppy playing with?
        Subject: He was playing with the red rubber ball.
        Interviewer: How are the puppy and the boy the same?
        Subject: Both of them each have a disabled part of their body.
        Interviewer: So in the movie, who is missing a leg?
        Interviewer: The boy, the puppy, both the boy and the puppy, or no one?
        Subject: The boy.
        Interviewer: Great.
        Interviewer: So we're going to watch a short clip from the movie and then we'll talk about it.
        Subject: Okay.
        clip: Whoa, cool.
        Interviewer: How do you think the puppy was feeling?
        Subject: Happy.
        Interviewer: How do you think the boy was feeling?
        Subject: He's feeling happy too.
        Interviewer: And how did you feel while you were watching that pot?
        Subject: I guess happy.
        Interviewer: Right.
        Interviewer: How do you think the puppy was feeling?
        Subject: Sad.
        Interviewer: How do you think the boy was feeling?
        Subject: He was feeling angry.
        Interviewer: And how did you feel while you were watching that mom?
        Subject: I didn't like it.
        Interviewer: How do you think the puppy was feeling?
        Subject: He didn't mind.
        Interviewer: How do you think the boy was feeling?
        Subject: He was still angry.
        Interviewer: And how did you feel while you were watching that pup?
        Subject: I feel bad for the puppy.
        Interviewer: Great.
        clip: Mom, I'll be outside.
        Interviewer: How do you think the puppy was feeling?
        Subject: Um, happy.
        Interviewer: How do you think the boy was feeling?
        Subject: He was feeling happy too.
        Interviewer: And how did you feel while you were watching this pot?
        Subject: I was happy.
        Interviewer: Great, thank you.

    (example 1 output):
        "I hope you enjoyed the last movie. Have you seen it before?" -> "I have at the mug MRI."
        "Can you tell me what happened in the movie? Try to tell the whole story. Remember that stories have a beginning,
things that happen, and an ending." -> "So there='s a boy who was disabled, and he didn't want to go outside. He wasn't
```

bioRxiv preprint doi: https://doi.org/10.1101/2025.10.20.682864; this version posted October 20, 2025. The copyright holder for this preprint (which was not certified by peer review) is the author/funder, who has granted bioRxiv a license to display the preprint in perpetuity. It is made available under aCC-BY 4.0 International license.

```
in the mood for anything except for his video games. And then like his mom was trying to like help him go like get some fresh air and then but like everything like his mom tried to do didn't work so then he then she bought um him a puppy so then he started like he didn't want like to see the puppy like anymore because then he realized he realized that he was also like disabled. But then he started having a liking to him because he felt like the puppy and him were like the same. So he started playing with the puppy more. So then they went outside and they started playing with the ball."
        "Do you remember anything else from the story?" -> "I think that's much it."
        "What are some of the things you liked about the movie?" -> "I liked how he had a good relationship with his doll."
        "What are some of the things you didn't like about the movie?" -> "I liked everything about the movie, but I was hoping it would be longer."
        "Who gave the boy a box" -> "His mother."
        "What was in the box?" -> "The puppy."
        "What was the boy doing before he got the box?" -> "He wasn't really paying attention to anything. He was glued to his video games."
        "What was the puppy playing with?" -> "He was playing with the red rubber ball."
        "How are the puppy and the boy the same?" -> "Both of them each have a disabled part of their body."
        "In the movie, who is missing a leg? The boy, the puppy, both the boy and the puppy, or no one?" ->  "The boy."
        "How do you think the puppy was feeling? (after watching first clip)" -> "Happy."
        "How do you think the boy was feeling? (after watching first clip)" -> "He's feeling happy too."
        "And how did you feel while you were watching that part? (after watching first clip)" -> "I guess happy."
        "How do you think the puppy was feeling? (after watching second clip)" -> "Sad."
        "How do you think the boy was feeling? (after watching second clip)" -> "He was feeling angry."
        "And how did you feel while you were watching that part? (after watching second clip)" -> "I didn't like it."
        "How do you think the puppy was feeling? (after watching third clip)" -> "He didn't mind."
        "How do you think the boy was feeling? (after watching third clip)" -> "He was still angry."
        "And how did you feel while you were watching that part? (after watching third clip)" -> "I feel bad for the puppy"
        "How do you think the puppy was feeling? (after watching fourth clip)" -> "Um, happy."
        "How do you think the boy was feeling? (after watching fourth clip)" -> "He was feeling happy too."
        "And how did you feel while you were watching that part? (after watching fourth clip)" -> "I was happy."
        "Rating" -> "1"

Example 2:

    (example 2 transcript):
        Interviewer: Okay, so I hope you enjoyed the last movie.
        Interviewer: Have you seen it before?
        Subject: No
        Interviewer: Um, so I'm going to need you to talk a little bit.
        Interviewer: Is that okay?
        Subject: Okay.
        Interviewer: Can you tell me what happened in the movie?
        Interviewer: Try to tell the whole story.
        Interviewer: Remember that stories have a beginning, things that happen, and an ending.
        Interviewer: Great.
        Interviewer: Do you remember anything else from the story?
        Interviewer: Okay.
        Interviewer: What are some of the things you liked about the movie?
        Interviewer: And what are some of the things you didn't like about the movie?
        Interviewer: So who gave the boy a box?
        Interviewer: What was in the box?
        Interviewer: What was the boy doing before he got the box?
        Interviewer: What was the puppy playing with?
        Interviewer: How are the puppy and the boy the same?
        Interviewer: Perfect.
        Interviewer: So in the movie, who is missing a leg?
        Interviewer: The boy, the puppy, both the boy and the puppy, or no one?
        Subject: Both the boy and the puppy.
```

bioRxiv preprint doi: https://doi.org/10.1101/2025.10.20.682864; this version posted October 20, 2025. The copyright holder for this preprint (which was not certified by peer review) is the author/funder, who has granted bioRxiv a license to display the preprint in perpetuity. It is made available under aCC-BY 4.0 International license.

```
        Interviewer: Great.
        Interviewer: So we're going to watch a couple of short clips from the movie and talk about them, OK?
        Subject: OK.
        clip: Whoa.
        clip: Cool.
        Interviewer: How do you think the puppy was feeling?
        Subject: Happy.
        Interviewer: How do you think the boy was feeling?
        Subject: Happy.
        Interviewer: Happy?
        Interviewer: And how did you feel while you were watching this part?
        Subject: Good.
        Interviewer: Good?
        Interviewer: Great.
        clip: You've got to be kidding me.
        Interviewer: How do you think the puppy was feeling?
        Interviewer: And how do you think the boy was feeling?
        Interviewer: And how did you feel while you were watching this part?
        clip: Get lost!
        Interviewer: How do you think the puppy was feeling?
        Interviewer: And how do you think the boy was feeling?
        Interviewer: And how did you feel while you were watching this pot?
        Interviewer: Okay, one more.
        clip: Mom!
        clip: We'll be outside!
        Interviewer: How do you think the puppy was feeling?
        Subject: Happy.
        Interviewer: How do you think the boy was feeling?
        Subject: Happy.
        Interviewer: And how did you feel while you were watching his paw?
        Subject: Happy.
        Interviewer: Great, thank you.


    (example 2 output):
        "I hope you enjoyed the last movie. Have you seen it before?" -> "No."
        "Can you tell me what happened in the movie? Try to tell the whole story. Remember that stories have a beginning, things that happen, and an ending." -> ""
        "Do you remember anything else from the story?" -> ""
        "What are some of the things you liked about the movie?" -> ""
        "What are some of the things you didn't like about the movie?" -> ""
        "Who gave the boy a box" -> ""
        "What was in the box?" -> ""
        "What was the boy doing before he got the box?" -> ""
        "What was the puppy playing with?" -> ""
        "How are the puppy and the boy the same?" -> ""
        "In the movie, who is missing a leg? The boy, the puppy, both the boy and the puppy, or no one?" ->  "Both the boy and the puppy."
        "How do you think the puppy was feeling? (after watching first clip)" -> "Happy."
        "How do you think the boy was feeling? (after watching first clip)" -> "Happy."
        "And how did you feel while you were watching that part? (after watching first clip)" -> "Good."
        "How do you think the puppy was feeling? (after watching second clip)" -> ""
        "How do you think the boy was feeling? (after watching second clip)" -> ""
        "And how did you feel while you were watching that part? (after watching second clip)" -> ""
        "How do you think the puppy was feeling? (after watching third clip)" -> ""
        "How do you think the boy was feeling? (after watching third clip)" -> ""
        "And how did you feel while you were watching that part? (after watching third clip)" -> ""
        "How do you think the puppy was feeling? (after watching fourth clip)" -> "Happy."
```

bioRxiv preprint doi: https://doi.org/10.1101/2025.10.20.682864; this version posted October 20, 2025. The copyright holder for this preprint (which was not certified by peer review) is the author/funder, who has granted bioRxiv a license to display the preprint in perpetuity. It is made available under aCC-BY 4.0 International license.

```
        "How do you think the boy was feeling? (after watching fourth clip)" -> "Happy."
        "And how did you feel while you were watching that part? (after watching fourth clip)" -> "Happy."
        "Rating" -> "0.1"

    (example 3 transcript):
        Interviewer: Have you seen that last movie before, the puppy one?
        Subject: I don't think I have.
        Interviewer: Can you try and tell me what happens in the movie?
        Interviewer: The full beginning, middle, and end.
        Subject: Um, the beginning, the beginning of the movie is, um, so it's the middle, mom gives him the puppy, but
he doesn't like it on first because it has three legs, and then towards the end,
        Subject: The last time I played with them.
        Subject: I don't know about it.
        Subject: All right, we both have the same disability.
        Subject: Okay.
        Subject: Didn't go home.
        Subject: How deep?
        Interviewer: What was the boy doing before he got the box?
        Interviewer: What was the puppy playing with?
        Interviewer: How are the puppy and the boy the same?
        Interviewer: In the movie, who's missing a leg?
        Interviewer: The boy, the puppy, both the boy and the puppy or no one?
        Interviewer: Okay, now we're going to watch a short clip and then we'll talk about it.
        Interviewer: How's the puppy feeling here?
        Interviewer: How's the boy feeling?
        Interviewer: And how do you feel when you watch his part?
        Interviewer: How's the puppy feeling here?
        Interviewer: How's the boy feeling?
        Interviewer: And how do you feel when you watch this part?
        Interviewer: How's the puppy feeling here?
        Interviewer: How's the boy feeling?
        Interviewer: How do you feel when you watch his fart?
        Interviewer: How's the puppy feeling here?
        Interviewer: How's the boy feeling?
        Interviewer: And how do you feeling off that part?
        Subject: Good.
        Interviewer: Okay, we're all done.

    (example 3 output):
        "I hope you enjoyed the last movie. Have you seen it before?" -> "I don't think I have."
        "Can you tell me what happened in the movie? Try to tell the whole story. Remember that stories have a beginning,
things that happen, and an ending." -> "Um, the beginning, the beginning of the movie is, um, so it's the middle, mom
gives him the puppy, but he doesn't like it on first because it has three legs, and then towards the end, The last time I
played with them. I don't know about it. All right, we both have the same disability. Okay. Didn't go home. How deep?"
        "Do you remember anything else from the story?" -> ""
        "What are some of the things you liked about the movie?" -> ""
        "What are some of the things you didn't like about the movie?" -> ""
        "Who gave the boy a box" -> ""
        "What was in the box?" -> ""
        "What was the boy doing before he got the box?" -> ""
        "What was the puppy playing with?" -> ""
        "How are the puppy and the boy the same?" -> ""
        "In the movie, who is missing a leg? The boy, the puppy, both the boy and the puppy, or no one?" ->  ""
        "How do you think the puppy was feeling? (after watching first clip)" -> ""
        "How do you think the boy was feeling? (after watching first clip)" -> ""
        "And how did you feel while you were watching that part? (after watching first clip)" -> ""
        "How do you think the puppy was feeling? (after watching second clip)" -> ""
        "How do you think the boy was feeling? (after watching second clip)" -> ""
```

bioRxiv preprint doi: https://doi.org/10.1101/2025.10.20.682864; this version posted October 20, 2025. The copyright holder for this preprint (which was not certified by peer review) is the author/funder, who has granted bioRxiv a license to display the preprint in perpetuity. It is made available under aCC-BY 4.0 International license.

```
        "And how did you feel while you were watching that part? (after watching second clip)" -> ""
        "How do you think the puppy was feeling? (after watching third clip)" -> ""
        "How do you think the boy was feeling? (after watching third clip)" -> ""
        "And how did you feel while you were watching that part? (after watching third clip)" -> ""
        "How do you think the puppy was feeling? (after watching fourth clip)" -> ""
        "How do you think the boy was feeling? (after watching fourth clip)" -> ""
        "And how did you feel while you were watching that part? (after watching fourth clip)" -> "Good."
        "Rating" -> "0"

    (example 4 transcript):
        Interviewer: I hope you enjoyed the last movie.
        Interviewer: Have you seen it before?
        Subject: Yeah.
        Subject: The puppy cartoon?
        Subject: Yeah.
        Interviewer: Can you tell me what happened in the movie and try to tell the whole story?
        Interviewer: Remember that stories have a beginning, things that happen, and an ending.
        Subject: So first there was a boy that was playing a game.
        Subject: So when his mother came in the door was a box.
        Subject: She put it down and then a puppy was in the box.
        Subject: But the boy didn't want to play.
        Subject: Instead he wanted to play on his TV.
        Subject: So first he just kicked the puppy, so then he just came back and he did it again.
        Subject: So then he just went crazy and then got a ball.
        Subject: And then he wanted to play, but then the boy just kicked the ball in the box and the puppy ran with it.
        Subject: And then
        Subject: he dropped the ball back.
        Subject: And then the boy stopped when he was playing, and then he went to lose the puppy.
        Interviewer: Do you remember anything else from the story?
        Subject: That's all I can remember.
        Interviewer: What are some of the things you liked about the movie?
        Subject: When you like
        Subject: And then all of the puppies ran with it and then he got trapped in the box.
        Subject: When he kicked the puppy.
        Subject: His mom.
        Subject: A puppy.
        Subject: Playing on his TV.
        Subject: A ball.
        Interviewer: How are the puppy and the boy the same?
        Subject: They're both playing with them.
        Interviewer: In the movie, who is missing a leg?
        Interviewer: The boy, the puppy, both the boy and the puppy, or no one?
        Subject: The boy and the puppy.
        Interviewer: Let's watch a short clip from the movie, and then we'll talk about it.
        Interviewer: Give me one second, bud.
        Subject: Can we do an interview on it?
        Interviewer: This is the interview.
        Interviewer: Okay, here we go.
        Interviewer: You can sit back.
        Interviewer: Can you sit in your chair?
        Interviewer: How was the puppy feeling?
        Subject: Happy.
        Interviewer: How was the boy feeling?
        Subject: Happy.
        Interviewer: How did you feel when you watched that part?
        Subject: Happy.
        Subject: Ugh.
        clip: You've just got to be kidding me.
```

bioRxiv preprint doi: https://doi.org/10.1101/2025.10.20.682864; this version posted October 20, 2025. The copyright holder for this preprint (which was not certified by peer review) is the author/funder, who has granted bioRxiv a license to display the preprint in perpetuity. It is made available under aCC-BY 4.0 International license.

```
        Interviewer: How was the puppy feeling?
        Subject: He was feeling bad.
        Interviewer: How was the boy feeling?
        Subject: He was feeling, um, angry.
        Interviewer: How did you feel when you watched this part?
        Subject: Bad.
        Subject: Sad.
        Subject: Angry.
        Subject: Boys.
        Subject: Something.
        Subject: Get the boy and the puppy.
        Subject: I miss the boy.
        Interviewer: How's the puppy feeling?
        Subject: Very happy.
        Interviewer: How's the boy feeling?
        Subject: Super happy.
        Interviewer: How did you feel while you were watching that part?
        Subject: Happy.
        Interviewer: That's all, thank you.

    (example 4 output):
        "I hope you enjoyed the last movie. Have you seen it before?" -> "Yeah, The puppy cartoon? Yeah."
        "Can you tell me what happened in the movie? Try to tell the whole story. Remember that stories have a beginning, things that happen, and an ending." -> "So first there was a boy that was playing a game. So when his mother came in the door was a box. She put it down and then a puppy was in the box. But the boy didn't want to play. Instead he wanted to play on his TV. So first he just kicked the puppy, so then he just came back and he did it again. So then he just went crazy and then got a ball. And then he wanted to play, but then the boy just kicked the ball in the box and the puppy ran with it. And then he dropped the ball back. And then the boy stopped when he was playing, and then he went to lose the puppy."
        "Do you remember anything else from the story?" -> "That's all I can remember."
        "What are some of the things you liked about the movie?" -> "When you like. And then all of the puppies ran with it and then he got trapped in the box."
        "What are some of the things you didn't like about the movie?" -> "When he kicked the puppy."
        "Who gave the boy a box" -> "His mom"
        "What was in the box?" -> "A puppy"
        "What was the boy doing before he got the box?" -> ""Playing on his TV."
        "What was the puppy playing with?" -> "A ball."
        "How are the puppy and the boy the same?" -> "They're both playing with them."
        "In the movie, who is missing a leg? The boy, the puppy, both the boy and the puppy, or no one?" ->  "The boy and the puppy."
        "How do you think the puppy was feeling? (after watching first clip)" -> "Happy."
        "How do you think the boy was feeling? (after watching first clip)" -> "Happy."
        "And how did you feel while you were watching that part? (after watching first clip)" -> "Happy. Ugh."
        "How do you think the puppy was feeling? (after watching second clip)" -> "He was feeling bad."
        "How do you think the boy was feeling? (after watching second clip)" -> "He was feeling, um, angry."
        "And how did you feel while you were watching that part? (after watching second clip)" -> "Bad."
        "How do you think the puppy was feeling? (after watching third clip)" -> "Sad."
        "How do you think the boy was feeling? (after watching third clip)" -> "Angry."
        "And how did you feel while you were watching that part? (after watching third clip)" -> ""
        "How do you think the puppy was feeling? (after watching fourth clip)" -> "Very happy."
        "How do you think the boy was feeling? (after watching fourth clip)" -> "Super happy."
        "And how did you feel while you were watching that part? (after watching fourth clip)" -> "Happy."
        "Rating" -> "0.8"


### This is the transcript you have to process:
<<<transcript>>>
"""
```
